# Supplementary material for: Impact of the Euro 2020 championship on the spread of COVID-19
Source: Nat Commun. 2023 Jan 18;14:122. doi: 10.1038/s41467-022-35512-x (PMC9849464; doi:10.1038/s41467-022-35512-x)
Supplement: Supplementary file 1 — Supplementary Information [file 41467_2022_35512_MOESM1_ESM.pdf]

# Supplementary Information of Impact of the Euro 2020 championship on the spread of COVID-19

**Jonas Dehning[1,¶], Sebastian B. Mohr[1,¶], Sebastian Contreras[1], Philipp Dönges[1], Emil Iftekhar[1], Oliver Schulz[2], Philip Bechtle[3*], and Viola Priesemann[1,4,5 †]**

[1]Max Planck Institute for Dynamics and Self-Organization, Am Faßberg 17, 37077 Göttingen, Germany.

[2]Max Planck Institute for Physics, Föhringer Ring 6, 80805 München, Germany

[3]Physikalisches Institut, Universität Bonn, Nußallee 12, 53115 Bonn, Germany

[4]Institute for the Dynamics of Complex Systems, University of Göttingen, Friedrich-Hund-Platz 1, 37077 Göttingen, Germany.

[5]Institute of Computer Science and Campus Institute Data Science, University of Göttingen, Goldschmidtstraße 7, 24118 Göttingen, Germany

¶ These authors contributed equally

## Contents

**S1 Data sources** 2

**S2 Supplementary analysis: our results in context** 3

**S3 Supplementary Tables** 5

**S4 Supplementary Figures** 8

S4.1 Model including the effect of stadiums . . . 13

S4.2 Testing the detection of a null-effect . . . 13

S4.3 Robustness of parameters . . . 15

S4.4 Further analyses . . . 22

S4.5 Posterior of parameters . . . 27

S4.6 Chain mixing of selected parameters . . . 41

*bechtle@physik.uni-bonn.de

†viola.priesemann@ds.mpg.de

## S1 Data sources

We used the daily COVID-19 case numbers, resolved by age and country, as reported publicly by the state health institute or equivalent of each country covered in this work. The data was retrieved either directly or taken from COVerAGE-DB [1]:

- Germany: Robert Koch Institut
  `https://www.arcgis.com/home/item.html?id=f10774f1c63e40168479a1feb6c7ca74`
- France: Santé publique France
  `https://www.data.gouv.fr/fr/datasets/taux-dincidence-de-lepidemie-de-covid-19`
- England: National Health Service
  `https://coronavirus.data.gov.uk/details/download`
- Scotland: Public Health Scotland
  `https://www.opendata.nhs.scot/dataset/covid-19-in-scotland`
- Austria: Österreichische Agentur für Gesundheit und Ernährungssicherheit GmbH
  `https://covid19-dashboard.ages.at/`
- Belgium: Sciensano
  `https://epistat.wiv-isp.be/covid/`
- The Czech Republic: Ministerstvo zdravotnictví
  `https://onemocneni-aktualne.mzcr.cz/covid-19`
- Italy: Istituto Superiore di Sanità
  *Aggregated by COVerAGE-DB from*
  `https://www.epicentro.iss.it/coronavirus/sars-cov-2-sorveglianza-dati`
- The Netherlands: National Institute for Public Health and the Environment
  `https://data.rivm.nl/covid-19/`
- Slovakia: The Institute for Healthcare Analyses (IZA) of the Ministry of Health
  *Aggregated by COVerAGE-DB from*
  `https://github.com/Institut-Zdravotnych-Analyz/covid19-data`
- Spain: Ministry of Public Health
  *Aggregated by COVerAGE-DB from*
  `https://cnecovid.isciii.es/covid19/`

To estimate the deaths associated with the Euro 2020 cases we calculate the case fatality risk by using the number of deaths and number of cases as reported by Our World in Data (OWD) [2].

For showcasing the stringency of governmental measures (panel C in Fig. S24-S36), we used data from the Oxford COVID-19 Government Response Tracker [3] and the public health and social measures (PHSM) severity index [4] from the World Health Organization (WHO). For our correlational analysis of cases and human mobility (Fig. 3B and S4), we used data from the COVID-19 Community Mobility Reports [5] provided by Google. For correlation with pre-Euro 2020 incidences (Fig.S6B) we use case numbers as reported by the Johns Hopkins University (JHU) [6]. Lastly, we used data from Google Trends [7] to investigate people's interest in the Euro 2020 (Fig. S20).

## S2 Supplementary analysis: our results in context

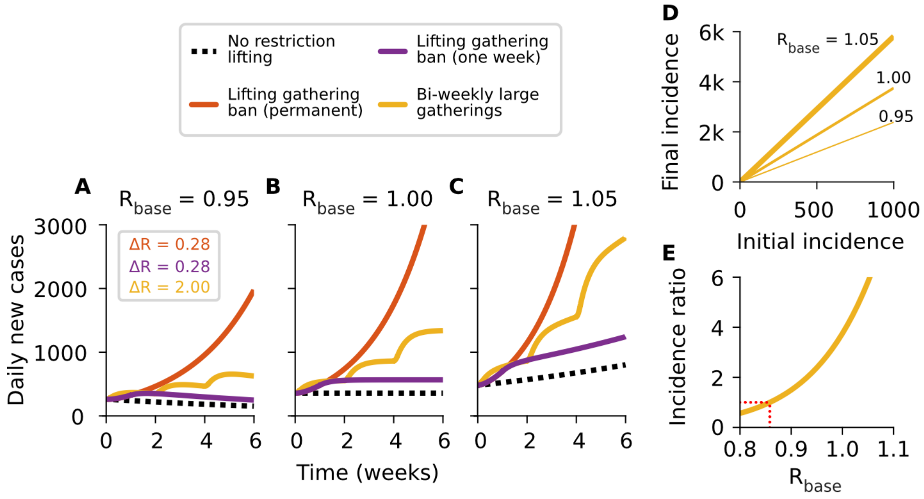


Supplementary Figure S1: **Our results in context: How much of an effect do short but strong increases of transmission have? A–C:** Understanding Euro 2020 matches as point interventions where the reproduction number is allowed to increase drastically from its base level $R_{\text{base}}$ for one day ($\Delta R = 2.0$, yellow curve), we compare its cumulative effect with different scenarios of lifting restrictions. These effects are in the order of magnitude of those reported in the literature [8]. The purple lines represent the same effect as a single increase but distributed over one week ($\Delta R = 0.28 \approx 2/7$), while the red curve represents a permanent lifting of those restrictions. The effect of the yellow and purple interventions is similar for $t \leq 2$ weeks because the product between $\Delta R$ and the duration of the intervention is the same. **D:** We observe long-term effects of consecutive interventions even when $R_{\text{base}}$ is lower than one (red dotted line). The impact of these effects increases exponentially with $R_{\text{base}}$. **E:** Similarly, the final incidence (after six weeks) increases with $R_{\text{base}}$. The red dotted line indicates that an incidence ratio larger than one can already result from values of $R_{\text{base}}$ smaller than one. Altogether, the cumulative effect of short but strong interventions (such as Euro 2020 matches) can be compared to lifting all bans on gatherings for a certain period of time. Curves were generated using a linear SEIS model without immunity for illustrative purposes.

To put our results in context, we compare the impact that different hypothetical scenarios of lifting of restrictions would have on case numbers (Fig. S1). Using a linear SEIS model for illustrative purposes, we evaluate three scenarios: i) Recurrent, bi-weekly (period $T = 2$ weeks) large events that strongly increase the reproduction number over its base level $R_{\text{base}}$ for one day by $\Delta R_s = 2.0$ (yellow curves). This effect size is comparable to what we inferred for some heated matches (e.g., Scotland - England for Scotland: $\Delta R_{\text{match}} = 3.5\,[2.9, 4.2]$, England - Italy for England: $\Delta R_{\text{match}} = 2.0\,[1.6, 3.5]$, England - Italy for Italy: $\Delta R_{\text{match}} = 0.9\,[-0.7, 4.4]$, and the Czech Republic - Denmark for the Czech Republic: $\Delta R_{\text{match}} = 2.7\,[0.8, 4.4]$). ii) A temporary one-week lifting of restrictions, with an effect equal to a single-day large event by distributing the increase in $R_{\text{base}}$ over a week: $\Delta R_w = 0.28 \approx 2/7$ (purple curves). iii) A permanent lifting of restrictions to the level of the second scenario: $\Delta R_p = 0.28$ for the considered time span (red curves). The value for $\Delta R_s$ in the first scenario is comparable to the largest effects found for the England-Scotland matches, while those in the second and third scenarios are similar to the effect of banning all private gatherings of 2 people or more as reported in [8].

The effect of interventions is comparable whenever the products between $\Delta R$ and the duration of the interventions are the same (e.g., yellow and purple curves for $t \leq 2$ weeks in Fig. S1A, B). In other words, the cumulative effect of short but strong interventions (such as Euro 2020 matches), can be compared to

lifting all bans on gatherings for a certain period of time. However, for regularly recurring interventions of size $\Delta R_s$, we observe permanent long-term effects when $R_{\text{base}} + \Delta R_s/T \geq 1$; the impact of recurring interventions increases disproportionately over time (Fig. S1A–C). Controlling the long-term effect of recurrent increases of the reproduction number is possible if the underlying reproduction number $R_{\text{base}}$ is small enough. Small changes of $R_{\text{base}}$ substantially impact the outcome, even below the $R_{\text{base}} = 1$ threshold, and in an exponential manner (Fig. S1D, E). This underlines the importance of control strategies if large-scale events are expected to temporally increase the spread of COVID-19.

On the other hand, quantitatively, the expected size $z$ of an infection chain depends on the effective reproduction number $R_{\text{eff}}$. As long as $R_{\text{eff}}$ is larger than one, the infection chains can become arbitrarily large. But even if $R_{\text{eff}} < 1$, one single infection is expected to cause $z = (1 - R_{\text{eff}})^{-1}$ infections before the chain dies out. For example, if $R_{\text{eff}} = 0.9$, a single infection caused by the Euro 2020 implies $z = 10$ infections in the total chain. Thus, in comparison, the primary cases have only a small contribution; the majority of the impact of an event like the Euro 2020 is the spread of subsequent infections into the general population (e.g., Fig. 2A).

## S3 Supplementary Tables

| Country | Median percentage of primary cases | Median percentage of subsequent cases | Median percentage of primary and subsequent cases | Probability that football increased cases |
|---|---|---|---|---|
| Avg. | 3.2% [1.3%, 5.2%] | - | - | > 99.9% |
| England | 12.4% [5.6%, 22.5%] | 36.0% [27.9%, 44.7%] | 47.8% [36.0%, 62.9%] | > 99.9% |
| Czech Republic | 9.7% [3.3%, 16.2%] | 47.8% [24.2%, 58.7%] | 57.7% [28.7%, 72.6%] | > 99.9% |
| Scotland | 3.3% [1.3%, 8.1%] | 36.6% [28.6%, 43.9%] | 40.8% [30.9%, 50.3%] | > 99.9% |
| Spain | 2.8% [-1.1%, 9.2%] | 24.1% [-16.3%, 60.6%] | 26.9% [-16.9%, 69.2%] | 91.8% |
| Italy | 2.1% [-5.8%, 10.9%] | 16.1% [-230.2%, 69.5%] | 18.7% [-235.6%, 78.4%] | 74.1% |
| Slovakia | 1.6% [-7.7%, 10.2%] | 15.5% [-88.2%, 50.6%] | 17.3% [-95.7%, 60.0%] | 70.8% |
| Germany | 1.4% [-1.8%, 4.2%] | 22.1% [-36.3%, 44.8%] | 23.6% [-38.0%, 48.6%] | 86.7% |
| Austria | 1.2% [-2.2%, 4.8%] | 24.0% [-62.9%, 60.8%] | 25.2% [-65.0%, 65.2%] | 79.4% |
| Belgium | 0.6% [-2.3%, 4.2%] | 9.2% [-60.0%, 47.9%] | 9.8% [-62.2%, 51.8%] | 67.6% |
| France | 0.5% [-0.2%, 1.4%] | 23.1% [-8.4%, 45.8%] | 23.6% [-8.6%, 47.0%] | 94.1% |
| Portugal | 0.3% [-2.6%, 2.7%] | -4.4% [-55.1%, 24.5%] | -4.1% [-57.4%, 26.9%] | 60.6% |
| The Netherlands | -1.5% [-3.3%, -0.2%] | -49.1% [-111.7%, -1.4%] | -50.6% [-114.6%, -1.7%] | 1.5% |

Supplementary Table S1: **Credible intervals from the posterior distribution** of the number of football related cases divided by the total number of cases during the championship. CI denotes 95% credible interval.

| Country | Primary cases per mil. people (male) | Primary cases per mil. people (female) | Primary and subsequent cases per mil. people |
|---|---|---|---|
| Avg. | - | - | 2228 [986, 3308] |
| England | 3595 [2661, 5729] | 1686 [1143, 3453] | 10600 [8185, 13875] |
| Czech Republic | 94 [40, 142] | 65 [22, 108] | 459 [229, 577] |
| Scotland | 1352 [940, 1758] | 351 [222, 517] | 7897 [6136, 9529] |
| Spain | 594 [-217, 1722] | 387 [-160, 1346] | 4518 [-2840, 11595] |
| Italy | 55 [-121, 227] | 27 [-77, 131] | 319 [-4001, 1335] |
| Slovakia | 8 [-30, 38] | 4 [-19, 25] | 57 [-313, 196] |
| Germany | 15 [-16, 36] | 7 [-11, 24] | 174 [-280, 359] |
| Austria | 42 [-70, 141] | 23 [-45, 100] | 642 [-1646, 1661] |
| Belgium | 34 [-112, 198] | 18 [-81, 155] | 411 [-2611, 2174] |
| France | 43 [-12, 95] | 27 [-8, 76] | 1515 [-552, 3008] |
| Portugal | 41 [-331, 340] | 25 [-247, 251] | -449 [-6294, 2960] |
| Netherlands | -186 [-328, -31] | -98 [-222, -13] | -4805 [-10851, -166] |

Supplementary Table S2: **Cases attributed to the Euro 2020 per million inhabitants** and related 95 % credible intervals in the male and female population. Primary and primary plus secondary cases are shown separately. Subsequent cases are almost gender-symmetric in all countries (see also Fig. S2). This indicates that also possible unobserved characteristics of the primary football-related infections in terms of other factors – such as age – are most likely distributed over the whole population in the course of subsequent infections.

| Country | Primary cases (male) | Primary cases (female) | Primary and subsequent cases | Estimated deaths associated with primary and subsequent cases |
|---|---|---|---|---|
| England | 93619 [69591, 145127] | 43872 [29946, 87030] | 567280 [436870, 747399] | 1227 [945, 1616] |
| Czech Republic | 494 [215, 753] | 346 [116, 558] | 4920 [2455, 6182] | 60 [30, 75] |
| Scotland | 3478 [2444, 4481] | 908 [574, 1320] | 41720 [31766, 50146] | 90 [69, 108] |
| Spain | 13570 [-4463, 40212] | 8870 [-3339, 31389] | 211952 [-122694, 546650] | 503 [-291, 1298] |
| Italy | 1535 [-3399, 6718] | 750 [-2219, 3824] | 17810 [-243916, 79338] | 170 [-2327, 757] |
| Slovakia | 21 [-87, 100] | 11 [-47, 67] | 320 [-1809, 1087] | 4 [-24, 14] |
| Germany | 618 [-629, 1460] | 306 [-440, 944] | 14626 [-23538, 29644] | 304 [-489, 616] |
| Austria | 178 [-308, 626] | 97 [-179, 436] | 6078 [-15534, 15387] | 34 [-86, 85] |
| Belgium | 191 [-600, 1091] | 101 [-441, 834] | 5352 [-31477, 24778] | 14 [-84, 66] |
| France | 1357 [-331, 2920] | 857 [-219, 2325] | 95929 [-40644, 190114] | 423 [-179, 838] |
| Portugal | 202 [-1683, 1667] | 122 [-1229, 1255] | -5205 [-72249, 29231] | -22 [-300, 121] |
| Netherlands | -1573 [-2756, -277] | -838 [-1859, -106] | -82805 [-181983, -3149] | -75 [-164, -3] |
| Total | 114769 [81915, 167796] | 56781 [36247, 100400] | 844609 [396860, 1253494] | 1689 [794, 2507] |

Supplementary Table S3: **Total cases attributed to the Euro 2020** and related 95 % credible intervals. The associated deaths are calculated under the assumption that the cases were equally distributed among age-groups and using the case fatality risk for the respective country in the time window of the Euro 2020.

| Country | $\Delta R_{\mathrm{match}}^{\mathrm{mean}}$ | Delay $D$ |
|---|---|---|
| Avg. | 0.46 [0.18, 0.75] | |
| England | 0.75 [0.01, 1.66] | 4.55 [4.36, 4.94] |
| Czech Republic | 1.26 [-0.50, 3.19] | 5.53 [4.75, 6.32] |
| Scotland | 1.09 [-2.77, 4.69] | 3.52 [3.35, 3.74] |
| Spain | 0.37 [-0.72, 1.83] | 6.91 [5.43, 7.82] |
| Italy | 0.28 [-1.11, 1.79] | 5.51 [3.96, 7.11] |
| Slovakia | 0.32 [-2.27, 2.56] | 5.00 [3.67, 7.28] |
| Germany | 0.33 [-0.62, 1.12] | 6.82 [5.69, 8.43] |
| Austria | 0.28 [-0.90, 1.45] | 4.58 [3.46, 6.37] |
| Belgium | 0.11 [-0.61, 0.92] | 5.09 [3.71, 6.69] |
| France | 0.30 [-0.46, 0.97] | 3.68 [3.13, 4.46] |
| Portugal | -0.02 [-1.33, 1.34] | 5.49 [4.30, 6.55] |
| Netherlands | -0.74 [-3.30, 1.36] | 5.70 [4.28, 6.00] |

Supplementary Table S4: **Average effect of Euro 2020 matches on the spread of COVID-19, per country.**

| Country | Matches played | Matches hosted | Union | Time between first and last match of the country (days) |
|---|---|---|---|---|
| England | 7 | 8 | 9 | 28 |
| Czech Republic | 5 | 0 | 5 | 19 |
| Scotland | 3 | 4 | 5 | 8 |
| Spain | 6 | 4 | 7 | 22 |
| Italy | 7 | 4 | 8 | 30 |
| Slovakia | 3 | 0 | 3 | 9 |
| Germany | 4 | 4 | 5 | 14 |
| Austria | 4 | 0 | 4 | 13 |
| Belgium | 5 | 0 | 5 | 20 |
| France | 4 | 0 | 4 | 13 |
| Portugal | 4 | 0 | 4 | 12 |
| Netherlands | 4 | 4 | 5 | 14 |

Supplementary Table S5: **Number of matches** played by the national team in the Euro 2020, matches played in the country and the union of the two categories. The union denotes the sum of the first two numbers without the overlapping matches.

## S4 Supplementary Figures

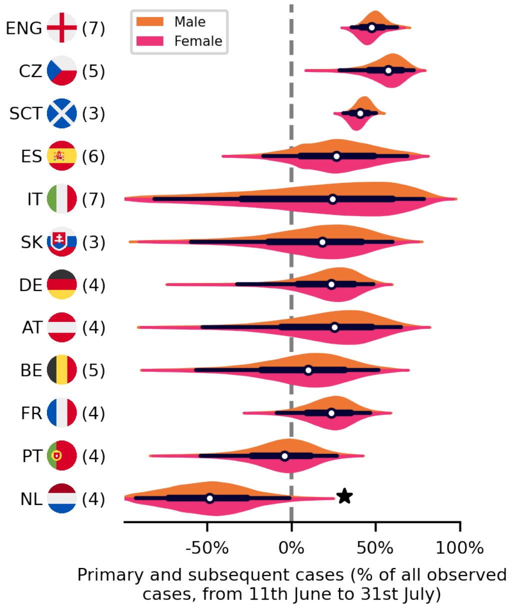


Supplementary Figure S2: **Overview of the sum of primary and subsequent cases accountable to the Euro 2020**. Calculations account for cases until July 31st, i.e., about three weeks after the championship finished. In the Netherlands ($\star$) the "freedom day" occurred on the same time as the Euro 2020. This effect also had a gender imbalance, thus, making it hard for our model to extract the Euro 2020 effect (see. Fig. S31). White dots represent median values, black bars and whiskers correspond to the 68% and 95% credible intervals (CI), respectively, and the distributions in color (truncated at 99% CI) represent the differences by gender ($n = 12$ countries).

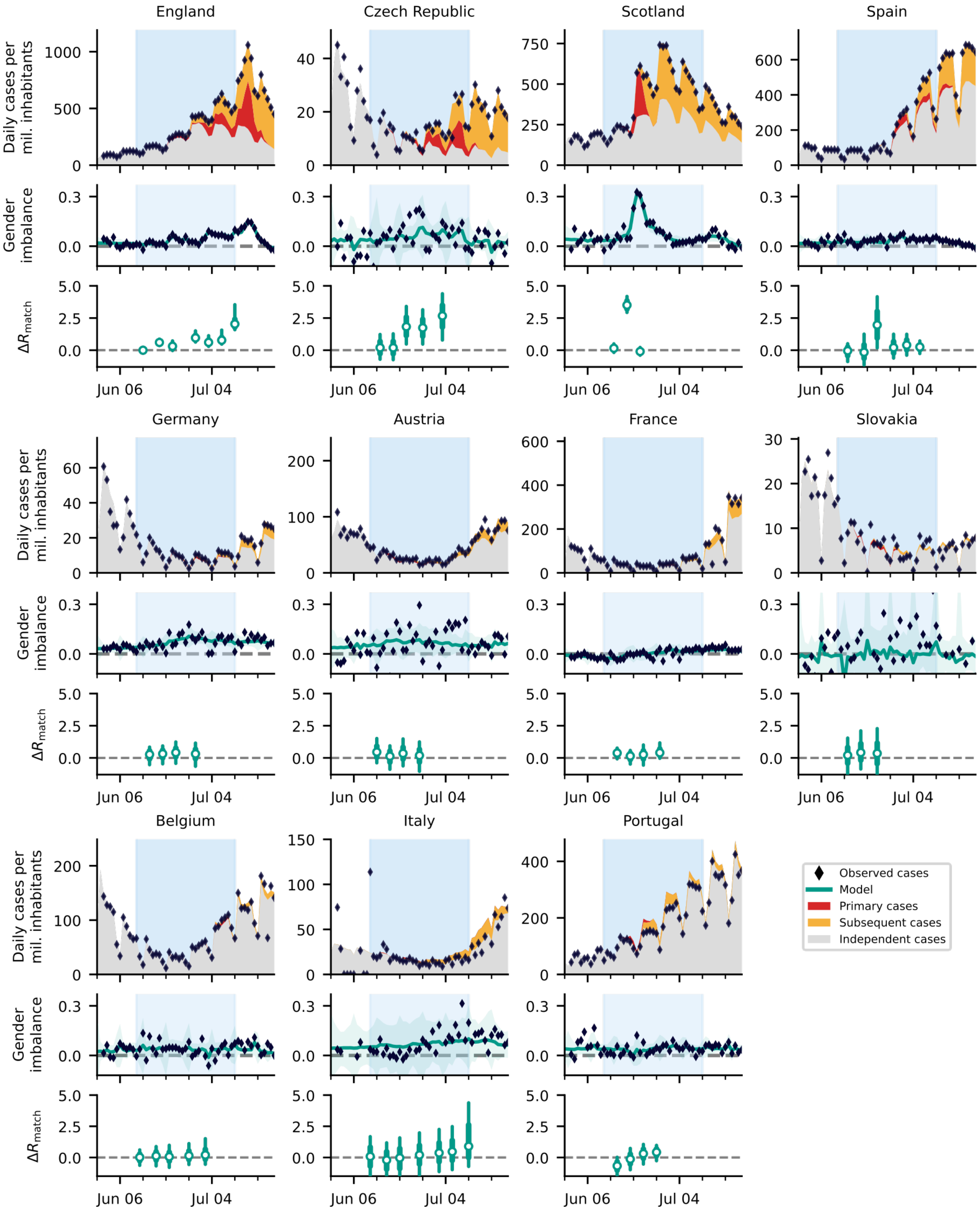


Supplementary Figure S3: **Overview of cases in all considered countries apart from the Netherlands** We split the observed incidence (black diamonds) of the three countries with the largest effect size into i) cases independent of Euro 2020 matches (gray area), ii) primary cases (directly associated with Euro 2020 matches, red area), and ii) subsequent cases (additional infection chains started by primary cases, orange area). See Figure 2 for more details. The turquoise shaded areas correspond to 95% CI. In the box plots, white dots represent median values, turquoise bars and whiskers correspond to the 68% and 95% credible intervals (CI), respectively.

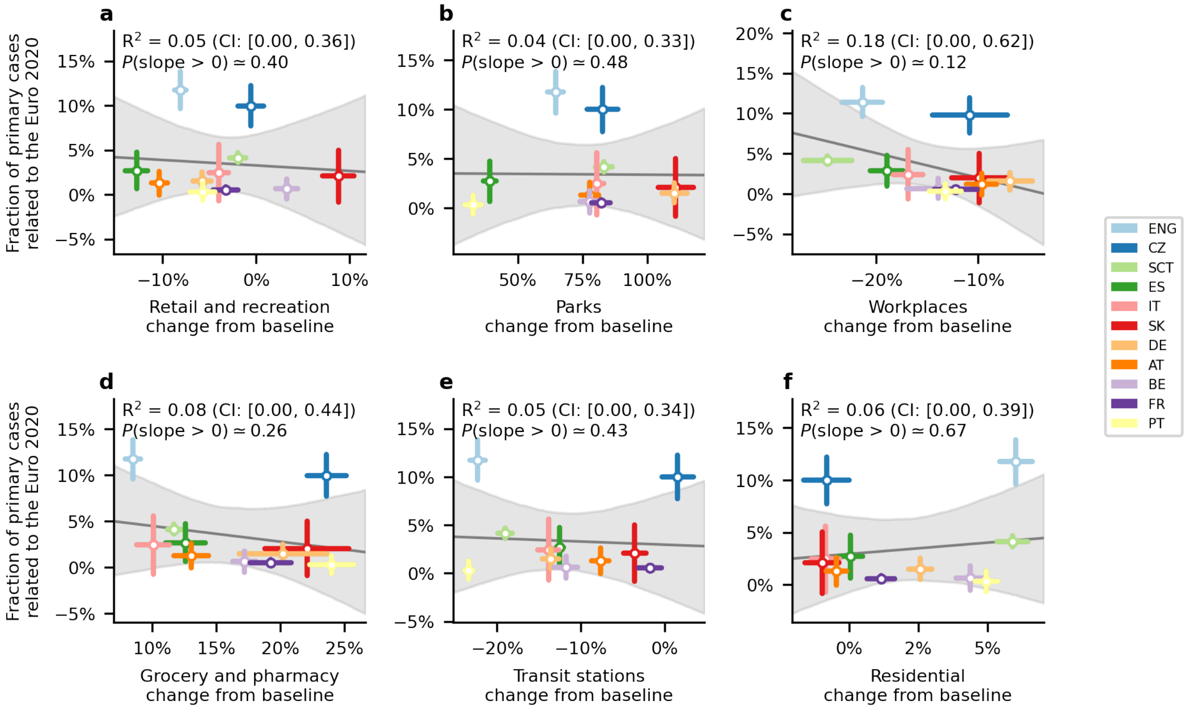


Supplementary Figure S4: **We found no significant correlation between cases arising from the Euro 2020 and human mobility.** Using mobility data from the "Google COVID-19 Community Mobility Reports" [5], we tested for correlation against the fraction of Euro 2020 related cases. Using the different categories (**A**-**F**) from the Mobility Report we found no significant correlation in either. The gray line and area are the median and 95% credible interval of the linear regression ($n = 11$ countries; The Netherlands was excluded for this analysis). Whiskers denote one standard deviation.

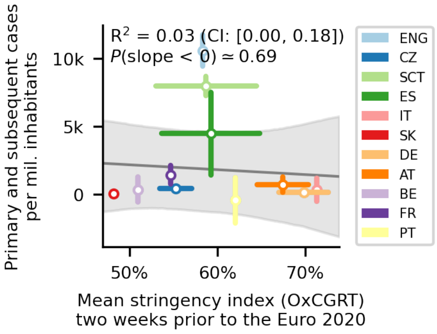


Supplementary Figure S5: **We found no significant correlation between cases arising from the Euro 2020 and the stringency of governmental interventions.** We correlated the average Oxford governmental response tracker [3] in the two weeks before the championship with the total number of cases per million inhabitants related to football gatherings. The gray line and area are the median and 95% credible interval of the linear regression ($n = 11$ countries; The Netherlands was excluded for this analysis). Whiskers denote one standard deviation.

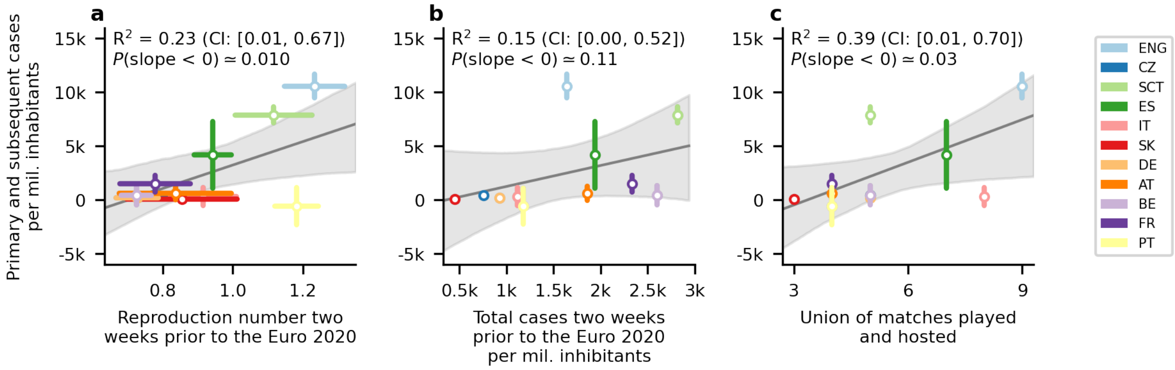


Supplementary Figure S6: **We found slight trends in the correlations between the impact of Euro 2020 and the base reproduction number and country popularity.** While these correlations are below the classical significance threshold of 0.05, they are less explanatory than the potential for spread (defined in Fig. 3). There was no significant correlation between the initial COVID-19 incidence and the impact of the Euro 2020. The gray line and area are the median and 95% credible intervals of the linear regression ($n$ = 11 countries; The Netherlands was excluded for this analysis). Whiskers denote one standard deviation.

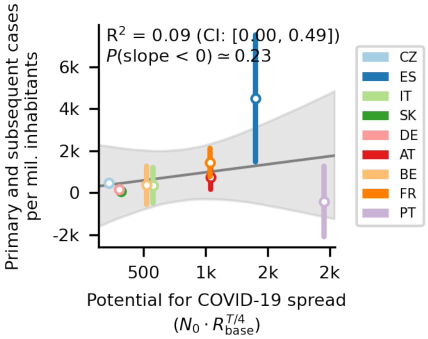


Supplementary Figure S7: **Prediction of the impact of Euro 2020 matches without the two most significant countries in the main model (England and Scotland).** The potential for spread, i.e., the number of COVID-19 cases that would be expected during the time $T$ a country is playing in the Euro 2020 ($N_0 \cdot R_{\mathrm{pre}}^{T/4}$) is still correlated with the number of Euro 2020-related cases after removing the two most significant entries from the analysis but not significantly. The observed slope without the most significant countries (median: 0.76, 95% CI: [-1.46, 3.04]) is consistent within its uncertainties with the slope including all countries (median: 1.62, 95% CI: [1.0, 2.26])). Due to the post-hoc nature of the removal of the most significant entries, this result is only shown for information. The gray line and area are the median and 95% credible interval of the linear regression ($n$ = 9 countries; The Netherlands, England and Scotland were excluded for this analysis). Whiskers denote one standard deviation.

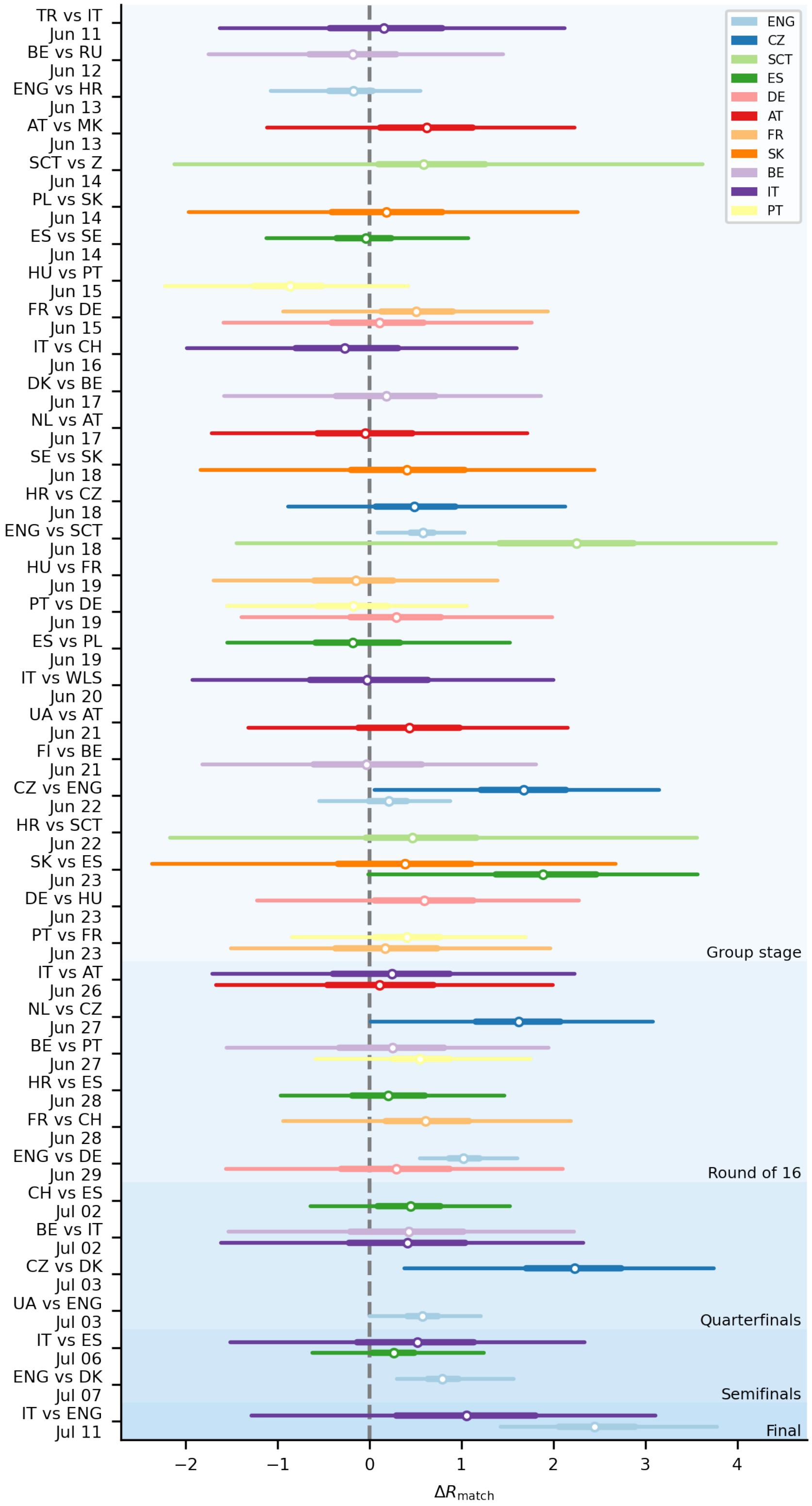


Supplementary Figure S8: **Effect of single Euro 2020 matches on the spread of COVID-19 across competing countries.** White dots represent median values, colored bars and whiskers correspond to the 68% and 95% credible intervals (CI).

### S4.1 Model including the effect of stadiums

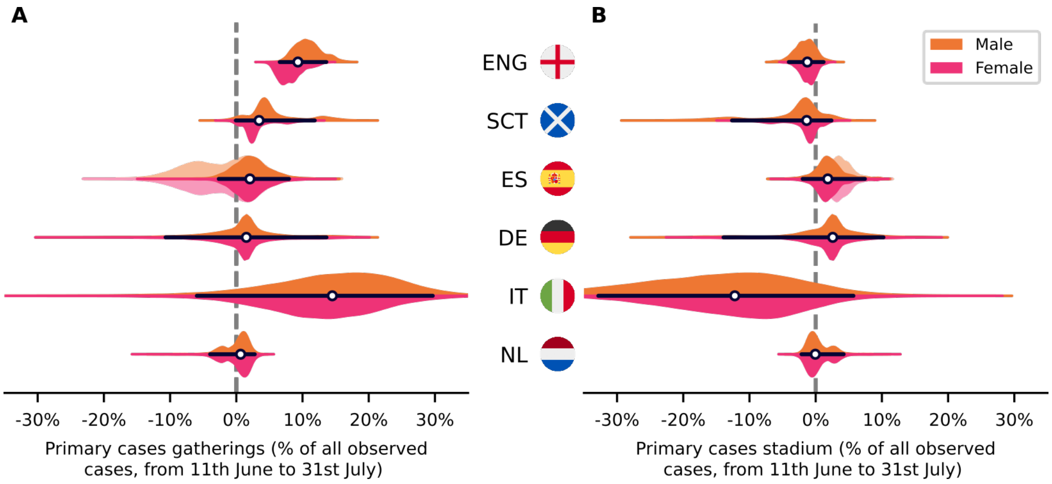


Supplementary Figure S9: **Including in our model the potential local transmission around the stadium where the matches occur does not significantly increase the overall effect.** In addition to the effect of football-related gatherings (**A**), we extended our model to include an additive effect on the reproduction number when a country hosted a match (**B**) (for those countries that hosted matches, i.e. $n = 6$ countries). We assume that local transmissions in and around the stadium would be detected mainly in the venue's country. However, football-related cases in a country where matches have a significant contribution to COVID-19 spread are tied to the dates of matches played by the country's team (A) and not to the country of the stadium venue (B), which is especially visible for England and Scotland. This also explains why previous attempts at measuring Euro 2020-related cases focusing on stadium venues were inconclusive. For Spain, an increase in the base reproduction number close to the date of a match makes the model inconclusive. In transparent is the region of the posterior of which we suppose that the model identifies the increase incorrectly; that is, where the posterior delay is smaller than 5.5 days. White dots represent median values, black bars and whiskers correspond to the 68% and 95% credible intervals (CI), respectively, and the distributions in color (truncated at 99% CI) represent the differences by gender.

### S4.2 Testing the detection of a null-effect

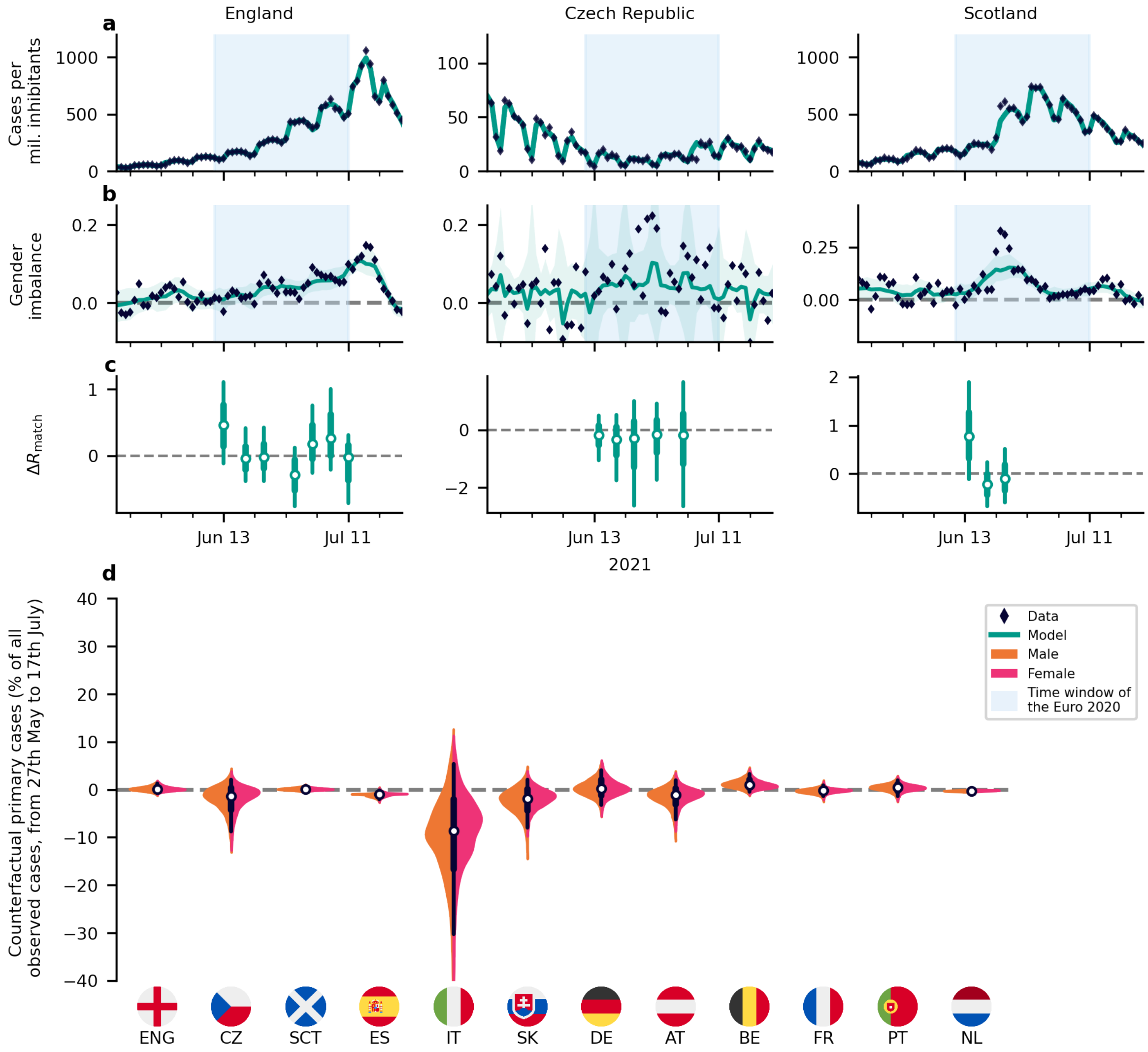


Supplementary Figure S10: **A temporal offset of 14 days leads to no inferred effect.** An artificial offset of the match data of 14 days decouples the gender ratio changes and the matches. This leads to no inferred effect of the championship – even in the three countries with the largest effect sizes in the main model (**A-C**). White dots represent median values, black bars and whiskers correspond to the 68% and 95% credible intervals (CI) ($n$ = 12 countries). Shaded turquoise area denotes 95% CI.

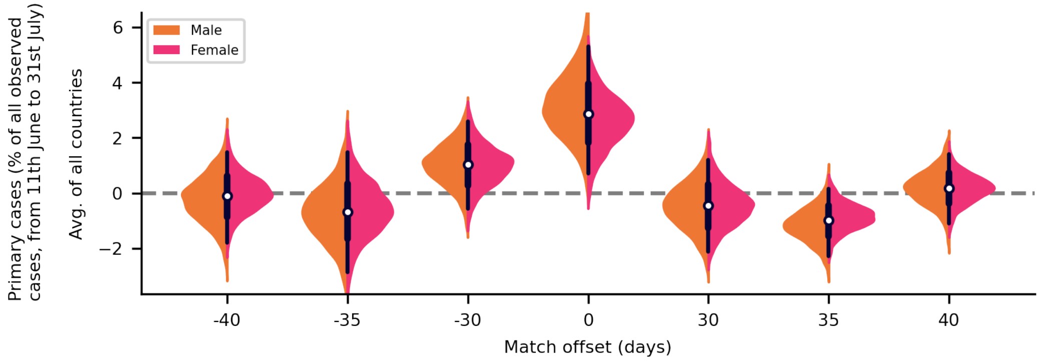


Supplementary Figure S11: **Changing the days of the match by a large offset results in a non-significant effect.** To test the reliability of our results, we ran counterfactual scenarios where the date of the matches was moved to lie outside the championship period. As expected, such offsets lead non-significant results of the average effect size across countries. White dots represent median values, black bars and whiskers correspond to the 68% and 95% credible intervals (CI) ($n = 11$ countries, The Netherlands was excluded for this analysis).

### S4.3 Robustness of parameters

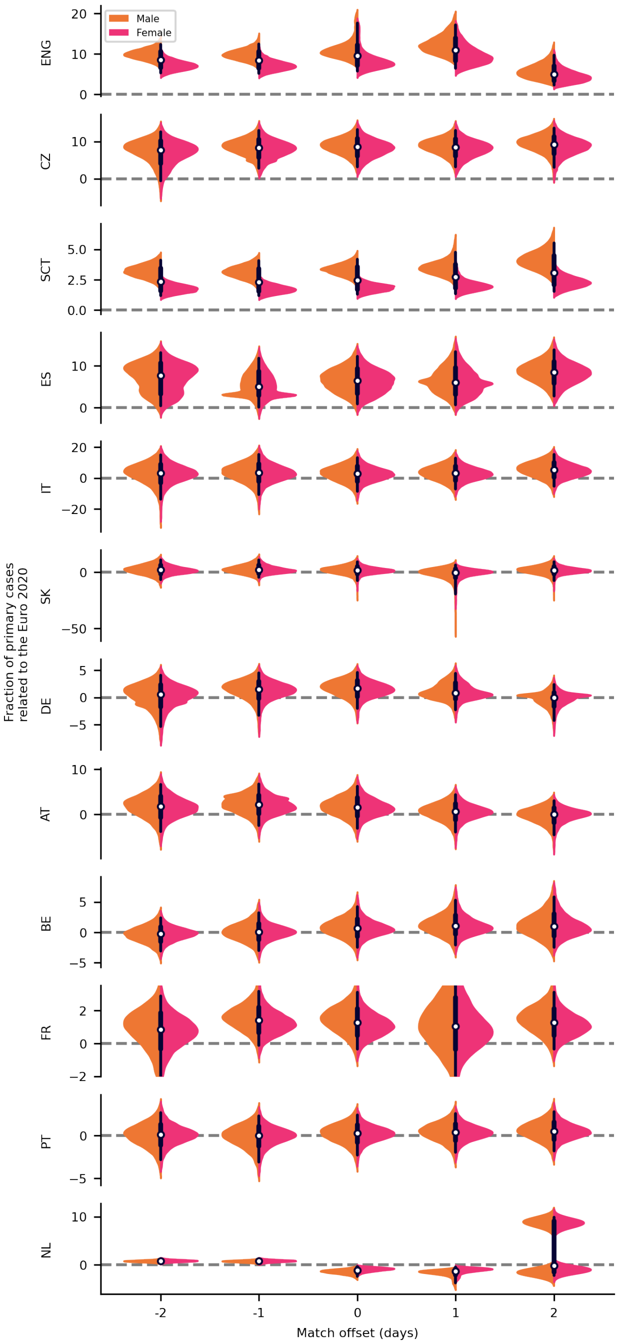


Supplementary Figure S12: **Robustness test for the effect of the temporal association between matches and cases by varying the effective delay.** We applied an artificial variation of all match days in a positive or negative direction. Under these relatively small variations of the delay, the gender imbalance is strong enough to lead to a stable effect size as the prior of the delay still allows for a sufficient shift of the posterior delay. The model run for France with a 1-day offset is missing because of an unknown, sampling-based error. White dots represent median values, black bars and whiskers correspond to the 68% and 95% credible intervals (CI), respectively, and the distributions in color (truncated at 99% CI) represent the differences by gender ($n = 11$ countries, The Netherlands was excluded for this analysis).

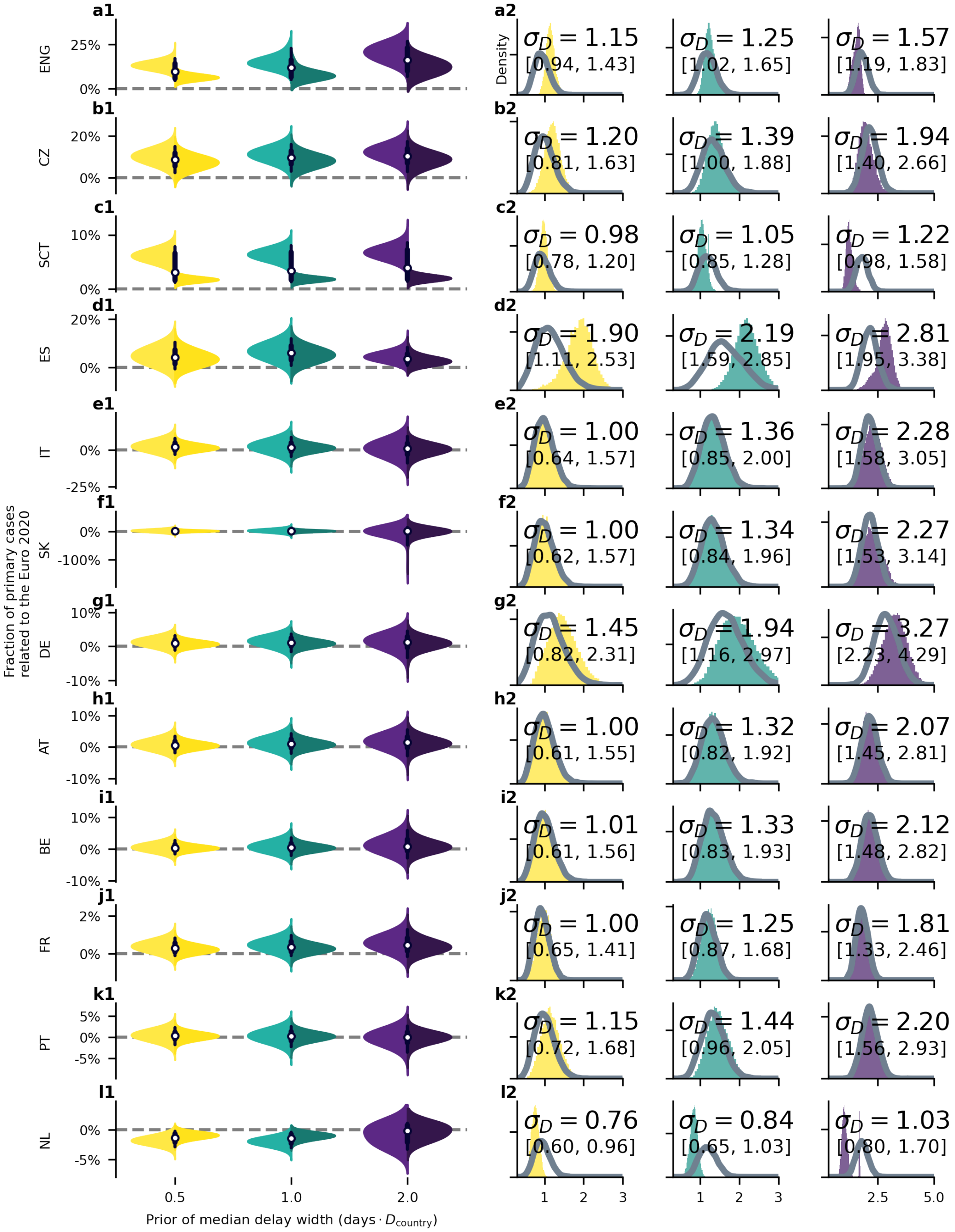

Supplementary Figure S13: **Robustness test for the effect of the width of the delay kernel.** In this robustness test, we varied the prior for the width of the delay kernel from the country-specific default (green) towards smaller (yellow) and larger (purple) widths (left column). In the violin plots, the left side is the prior for men; the right side for women. The right column shows the priors and resulting posterior of the standard deviation of the delay kernel $\sigma_D$. Except for England and Scotland (**A2, D2**), the data does not constrain this parameter. The results are not significantly modified in any country by changing the prior assumptions on this parameter (left column). On average, allowing for larger widths increases the effect size over the reported results. White dots represent median values, black bars and whiskers correspond to the 68% and 95% credible intervals (CI), respectively, and the distributions in color (truncated at 99% CI) represent the differences by gender.

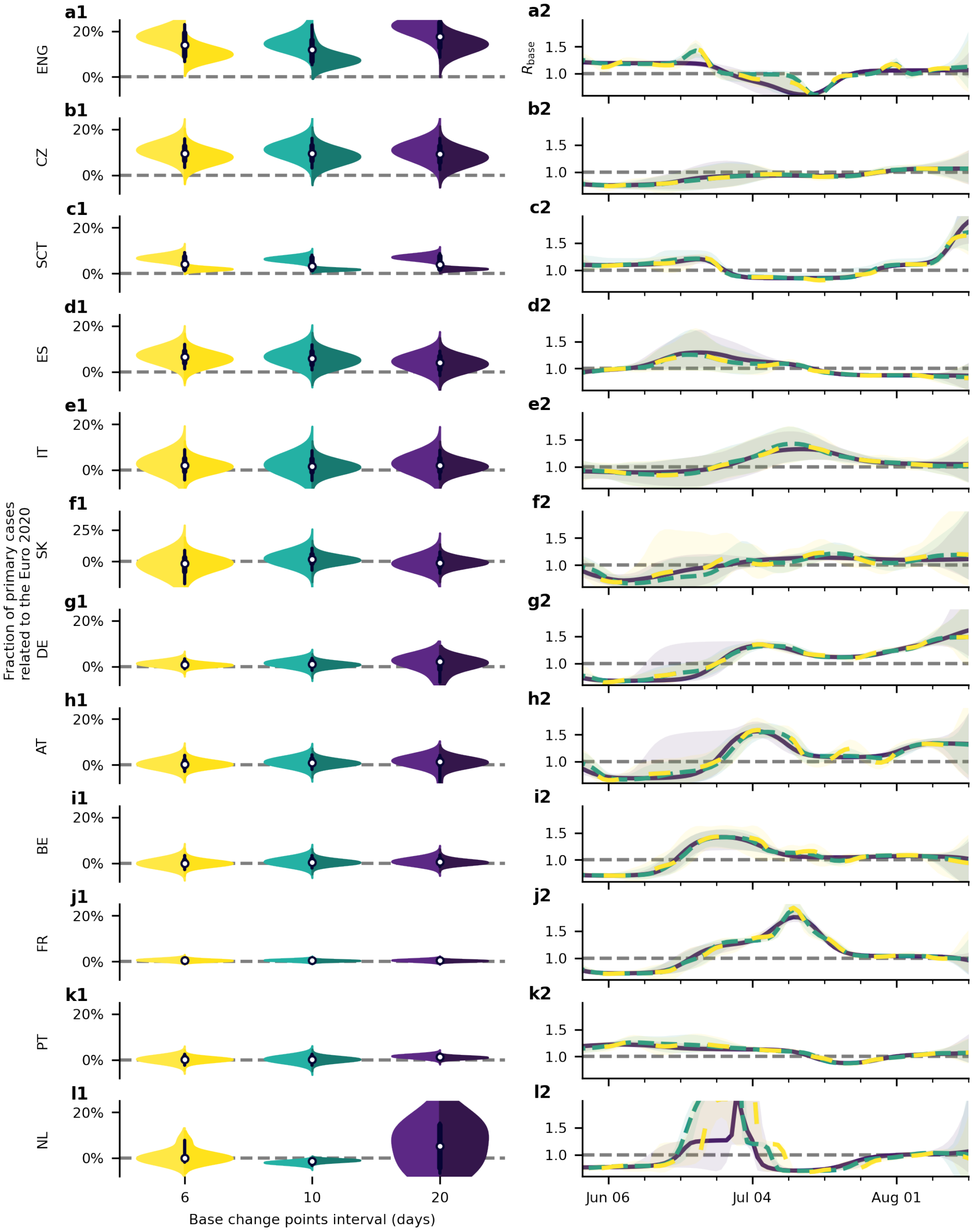


Supplementary Figure S14: **Robustness test for the effect of the allowed base reproduction number variability.** We propose models with three different base change point intervals: 6 days (yellow), 10 days (green), and 20 days (purple). In the violin plots, the left side is the distribution for men; the right side for women. We do not find significant differences in the fraction of football-related cases (left column) nor in the base reproduction numbers $R_{\text{base}}$ (right column). On average, allowing less variation in $R_{\text{base}}$ – i.e., removing the freedom of the model to absorb potential gender-symmetric and non-time-resolved cases related to football matches into short-timescale variations of $R_{\text{base}}$ – increases the effect size over the reported baseline results. Shaded areas in panels ***2** correspond to 95% CI. White dots represent median values, black bars and whiskers correspond to the 68% and 95% credible intervals (CI), respectively, and the distributions in color (truncated at 99% CI) represent the differences by gender.

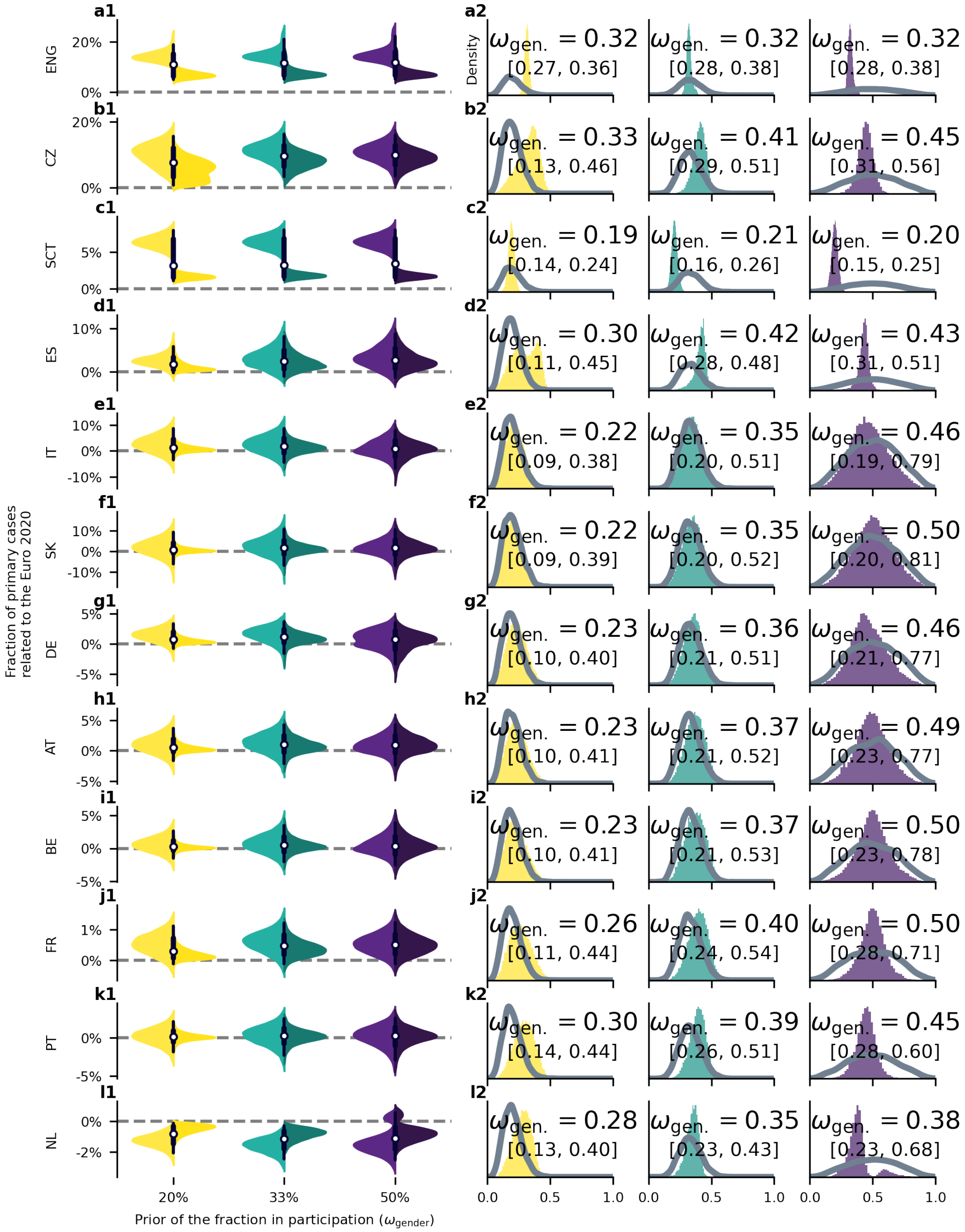


Supplementary Figure S15: **Robustness test for the effect of the fraction of female participation in football related gatherings** The default model employs a relatively constraining prior for the fraction of female participation in football-related gatherings (green) motivated by [9]. To check for the influence of this assumption, in an alternative model, we assume a more uninformative prior with mean female participation of 50% participation (purple) instead of 20% (green) (**A2-G2**). We do not find large differences in the results. On average, the total fraction of cases attributed to football matches grows when allowing the assumption of larger female participation in the fan gatherings. Hence, more cases are attributed to the Euro 2020 overall than in the baseline model. At the same time, a constraint used by the model for associating cases and matches is relieved. Thus, on average, the uncertainty of the posterior slightly grows (**A1-G1**). White dots represent median values, black bars and whiskers correspond to the 68% and 95% credible intervals (CI), respectively, and the distributions in color (truncated at 99% CI) represent the differences by gender.

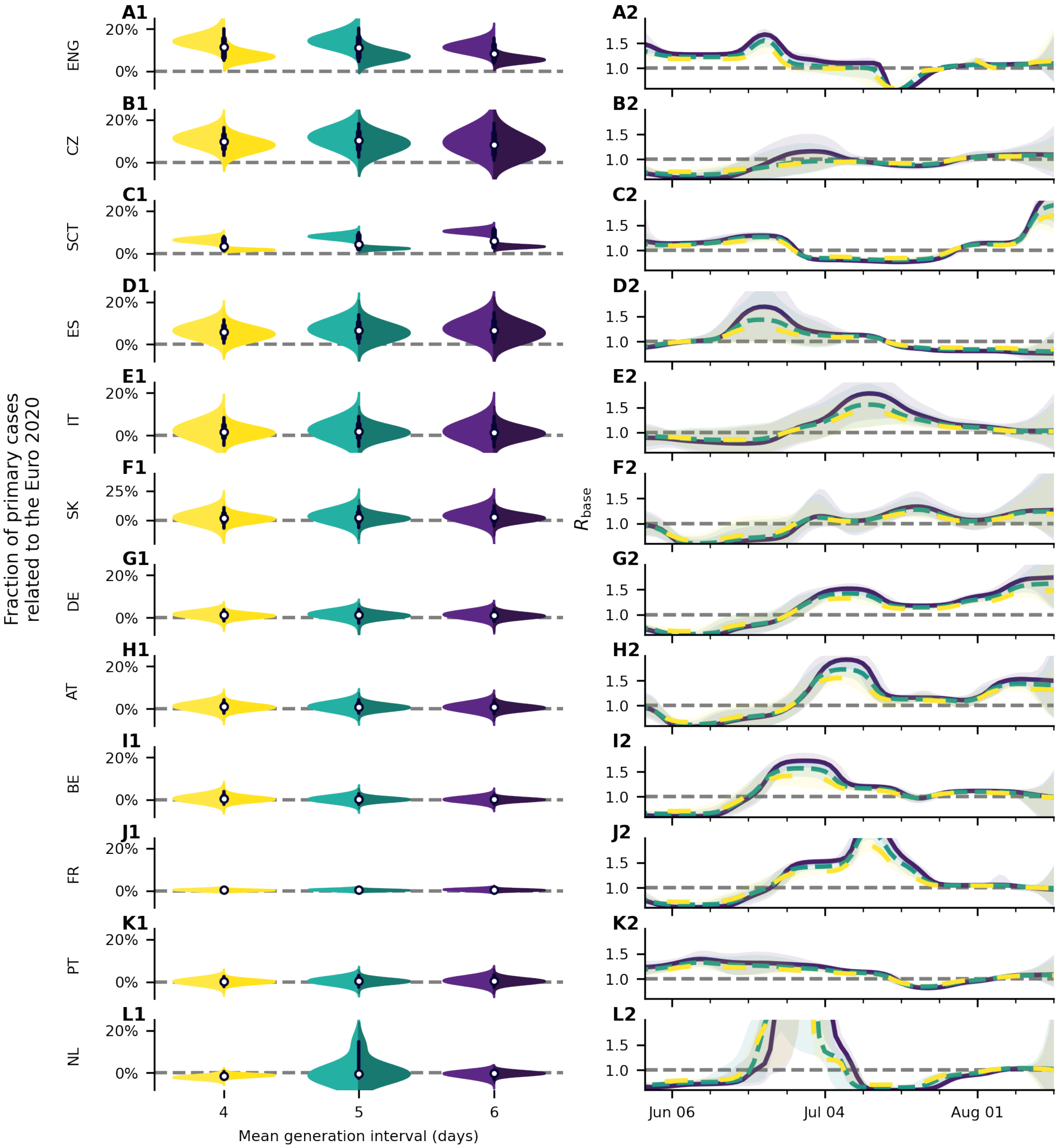


Supplementary Figure S16: **Robustness test for the effect the generation interval.** We propose models with three different generation intervals: with a mean of 4 days (yellow), 5 days (green), and 6 days (purple). The lack of significant difference in the fraction of football-related cases (left column) shows that if we assume a longer generation intervals than our base assumption of 4 days our conclusions do not change. One remarks that the the base reproduction numbers $R_{\text{base}}$ (right column) increases with a longer assumed generation interval, which is expected because a the increase of cases that needs to be modeled stays fixed. In the violin plots, the left side is the distribution for men; the right side for women. Shaded areas in the right column correspond to 95% CI. White dots represent median values, black bars and whiskers correspond to the 68% and 95% credible intervals (CI), respectively, and the distributions in color (truncated at 99% CI) represent the differences by gender.

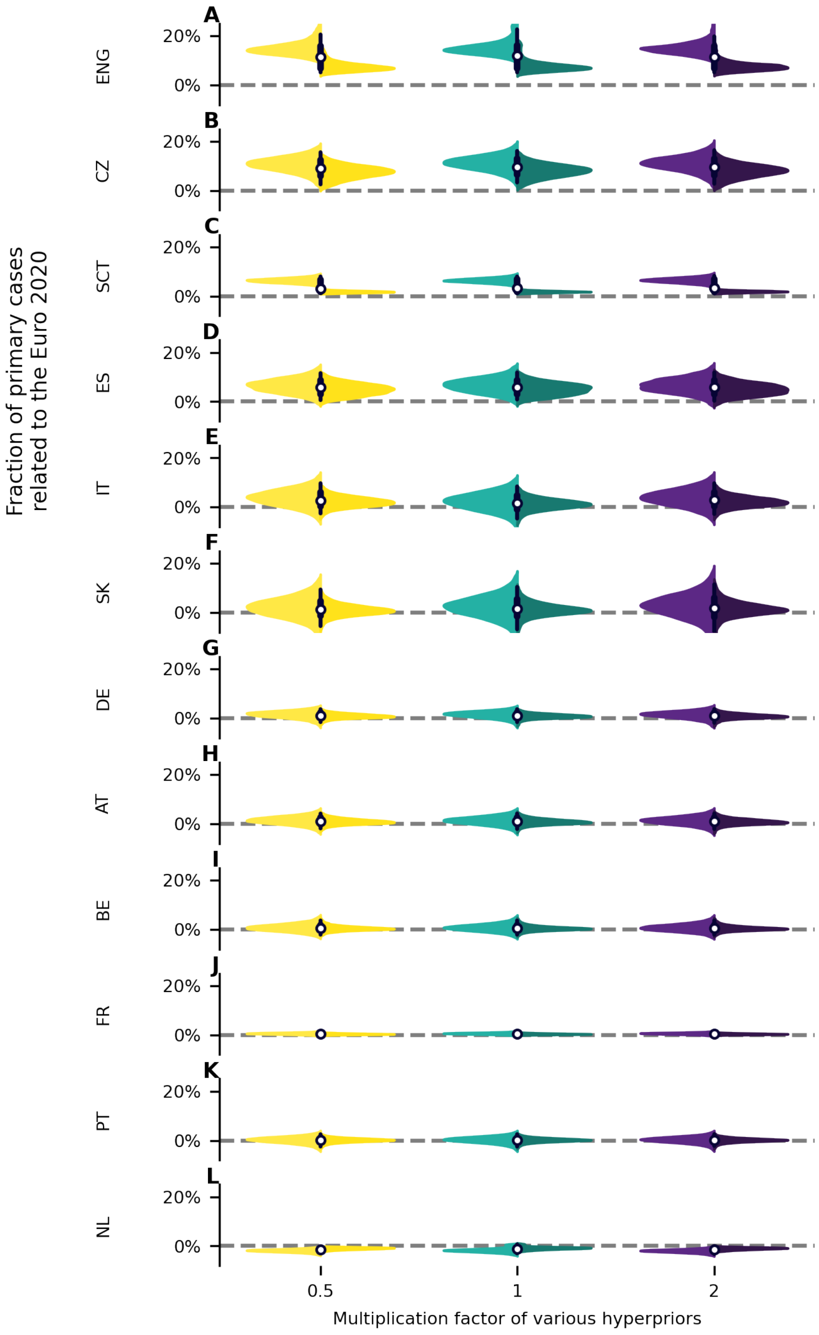


Supplementary Figure S17: **Robustness test for the remaining priors not studied in the previous figures.** Many of the priors in the model are relatively uninformative for the model. In these runs, we increased and decreased the prior value of the equations (16), (26), (35), (51), (52) and (54) by a factor of 2. In the violin plots, the left side is the distribution for men; the right side for women. White dots represent median values, black bars and whiskers correspond to the 68% and 95% credible intervals (CI), respectively, and the distributions in color (truncated at 99% CI) represent the differences by gender.

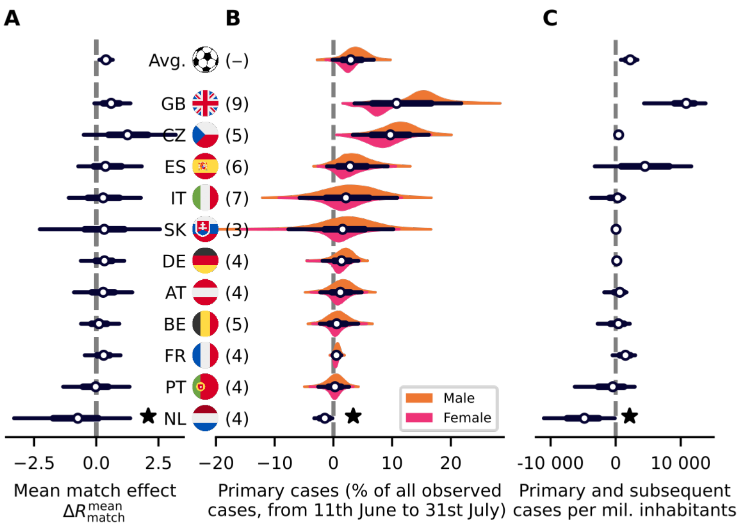


Supplementary Figure S18: **The combination of the case numbers of England and Scotland leads to similar results.** Because England and Scotland had each a team participating in the Euro 2020 we analyzed them separately, even if both are part of the United Kingdom. Here we added the case numbers of both (denoted as GB) and combined their matches for a new model run. The overall results do not change much in this alternative parametrization. White dots represent median values, black bars and whiskers correspond to the 68% and 95% credible intervals (CI), respectively, and the distributions in color (truncated at 99% CI) represent the differences by gender ($n = 11$ countries).

## S4.4 Further analyses

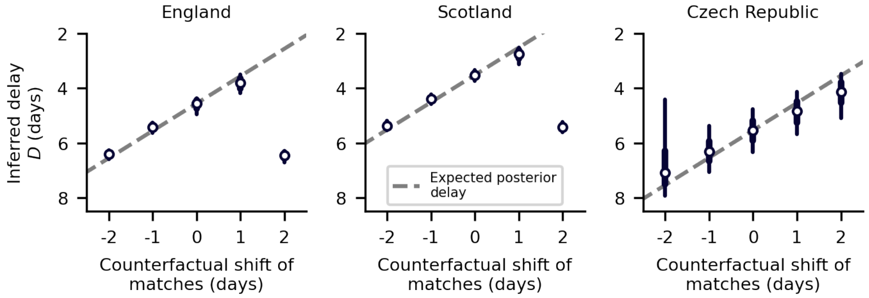


Supplementary Figure S19: **Our model is able to identify the delay between infection and reporting of it.** We tested counterfactual scenarios for England, Scotland and the Czech Republic where the dates of the matches were changed. Despite the same prior delay, the model managed to adapt the inferred delay to match the expected delay from the original model. White dots represent median values, black bars and whiskers correspond to the 68% and 95% credible intervals (CI).

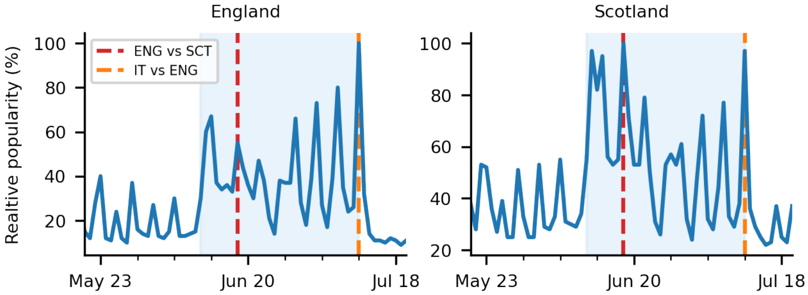


Supplementary Figure S20: **Relative popularity of the search term "football" in England and Scotland** measured using "Google Trends" [7] in the category "sport news". Vertical red lines represent the final and match of Scotland vs England, respectively.

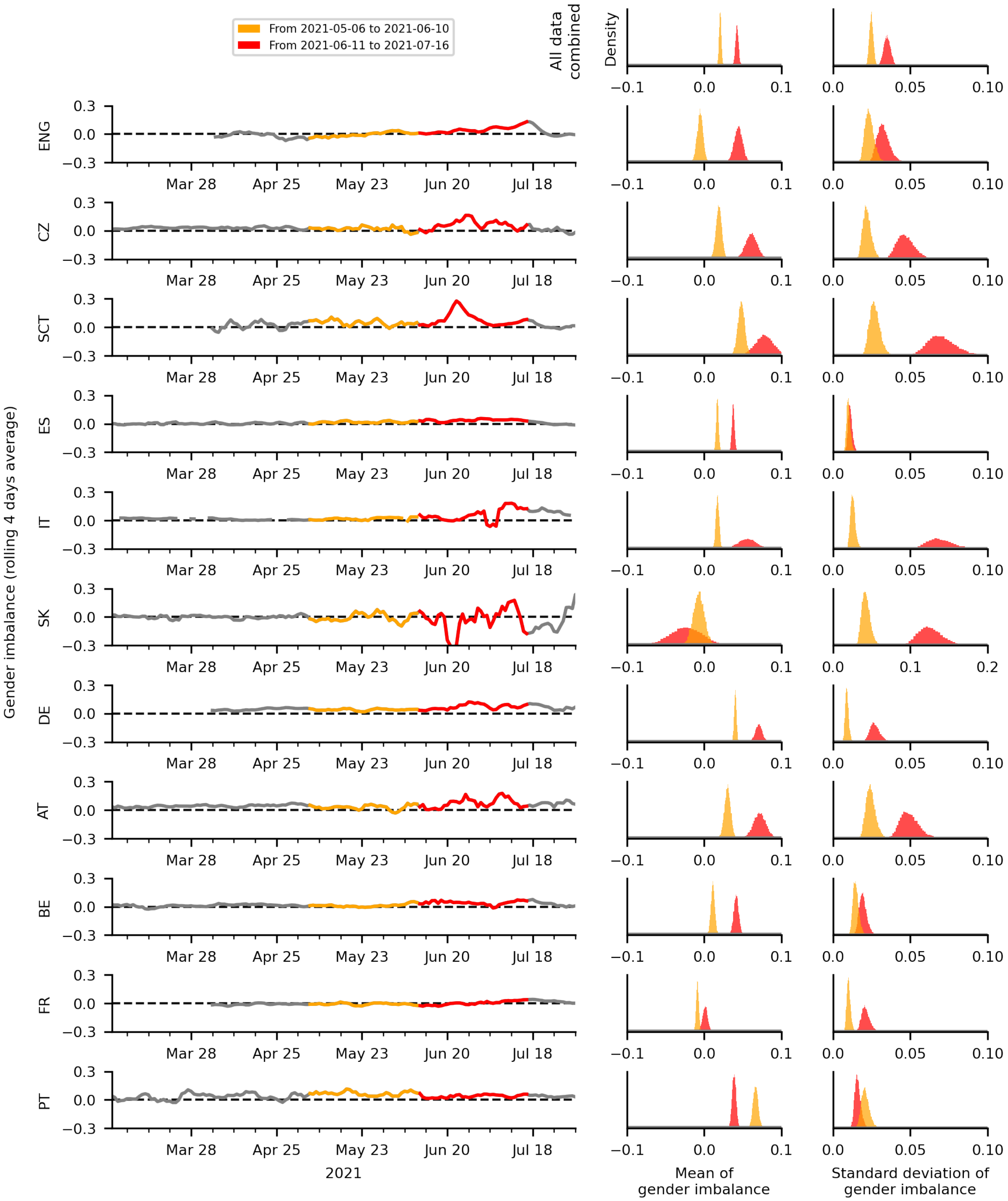


Supplementary Figure S21: **Male-female imbalance over time shows the largest deviations during championship.** We plotted the gender imbalance directly from our data (left column). All countries which showed significant effects had their largest imbalance change during or slightly after the championship (red), and also a number of non-significant countries display this behavior. In addition, the standard deviation of the imbalance during the championship (red) was on average larger than before the championship (orange, right column). This indicates that the large changes in imbalance during the championship were highly unusual and can't be attributed to chance alone. The red time period are the 30 days of the tournament plus the 5 days after and the orange time period the ones up to 35 days before the tournament.

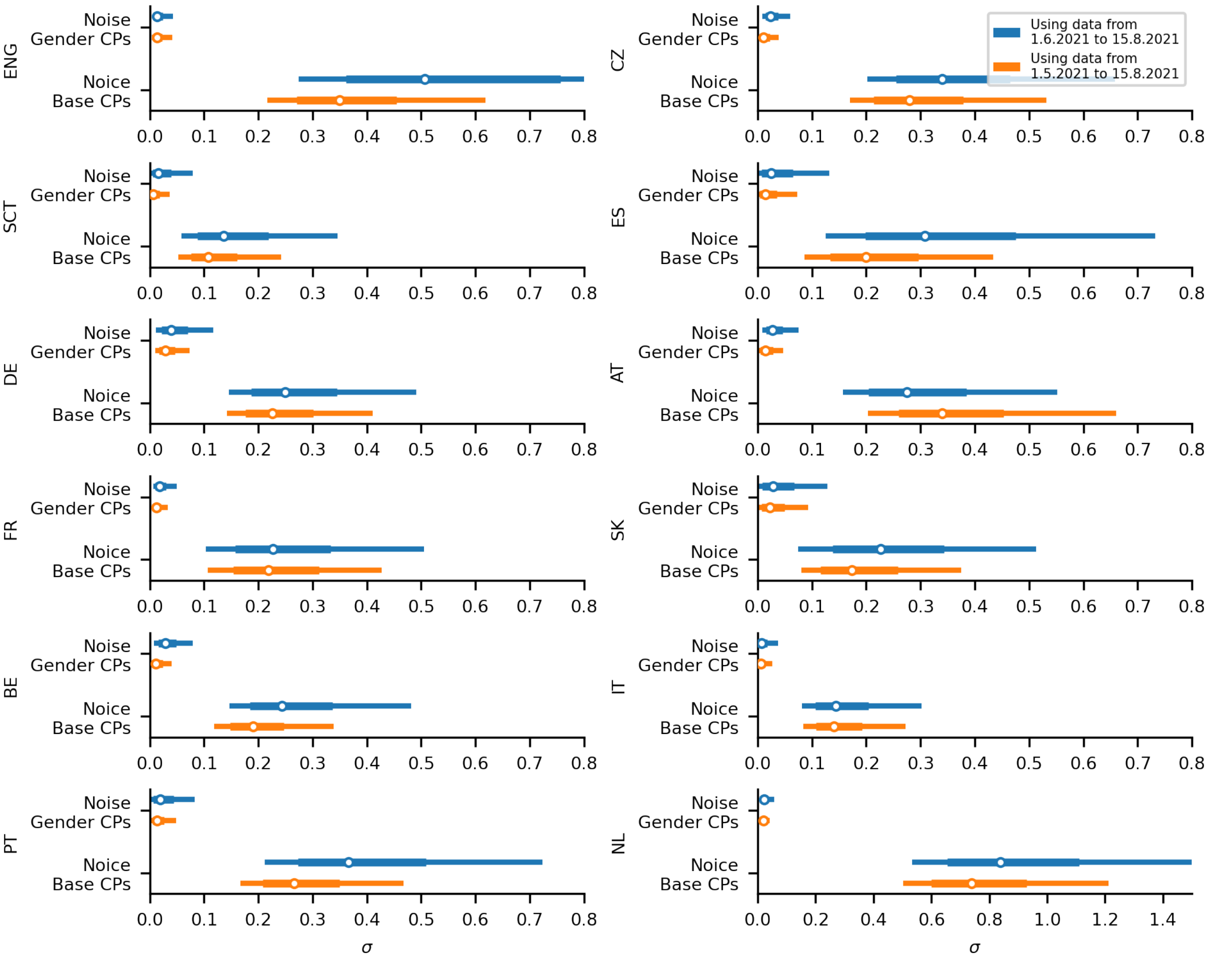


Supplementary Figure S22: **The inferred noise terms do not depend strongly on the length of the analyzed time-period.** We plotted the size of our gender noise term $\sigma_{\Delta\tilde{\gamma}}$ and the size of the change-points of the base reproduction number $\sigma_{\Delta\gamma}$. When beginning the run of our model a month earlier (blue), the noise terms do not change significantly compared to our base model (orange). White dots represent median values, colored bars and whiskers correspond to the 68% and 95% credible intervals (CI).

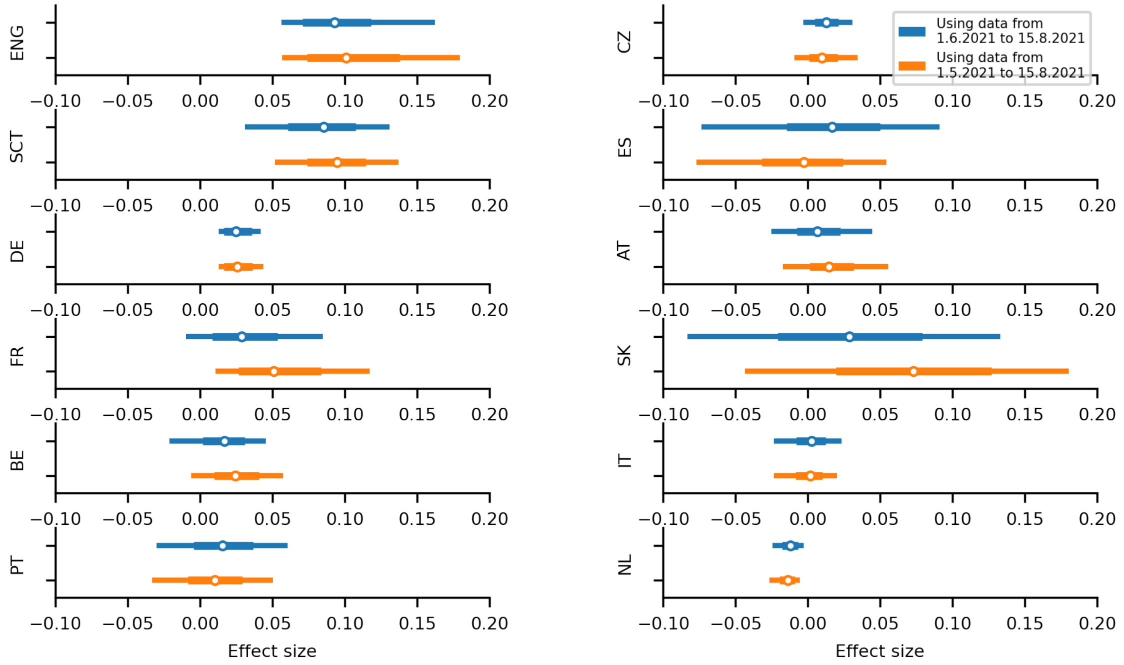


Supplementary Figure S23: **The inferred effect size (percentage of football-related primary infections) do not depend strongly on the length of the analyzed time-period.** To showcase that the total length of the analyzed period doesn't change significantly our results, we compare the percentage of football-related primary infections one-month-longer runs (blue) compared to our base model (orange). White dots represent median values, colored bars and whiskers correspond to the 68% and 95% credible intervals (CI).

### S4.5 Posterior of parameters

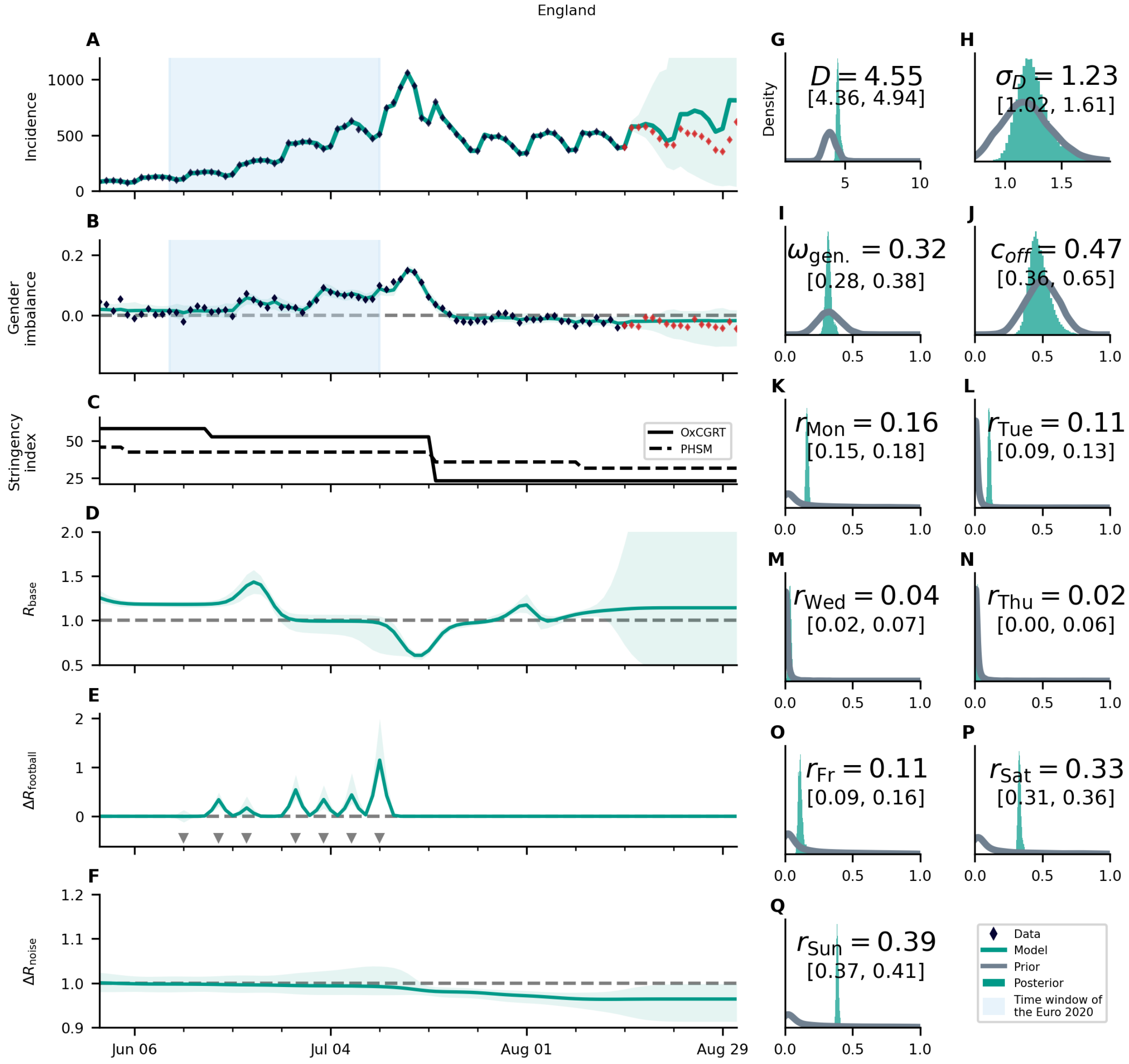


Supplementary Figure S24: **Overview of the posterior for England.** We compare (**A**) the time-dependence of the incidence before, during (blue shaded area) and after the championship; (**B**) the gender imbalance of observed cases; (**C**) Oxford governmental response tracker (OxCGRT) [3] and public health and social measures severity index (PHSM) [4] (not part of the model); (**D**) the gender-symmetric base reproduction number $R_{\text{base}}$; (**E**) the gender-asymmetric football reproduction number $R_{\text{football}}$; (**F**) gender-asymmetric noise related reproduction number $R_{\text{noise}}$; and (**G**) to (**Q**) the prior and posterior of various parameters. In mid July the incidence starts dropping. In contrast, the number of deaths continues to increase. Together, this indicates that the testing policy was changed around that time. England is one of the two countries where the delay $D$ and the female participation in fan activities dominating the additional transmission can be measured and significantly constrained with the data compared to the prior distribution (**G** and **I**). Red diamonds show data not used for the analysis. This comes with an increase in the uncertainty in the model prediction. One notes two slight bumps of the base reproduction number: one during and one after the end of the championship. The first bump may indicate that our model is not able to fully attribute a part of the effective reproduction number to $\Delta R_{\text{football}}$ and is attributing the effect of England's matches in the group phase to the base reproduction number instead. The second bump might be explained hereby: During the championship there may be generally more social contacts, which are not in temporal synchronization with the matches, and therefore not explained by $\Delta R_{\text{football}}$ but by $R_{\text{base}}$ instead. Hence, after the championship the base reproduction number decreases and increases again when measures are lifted (**C**). The turquoise shaded areas correspond to 95% credible intervals.

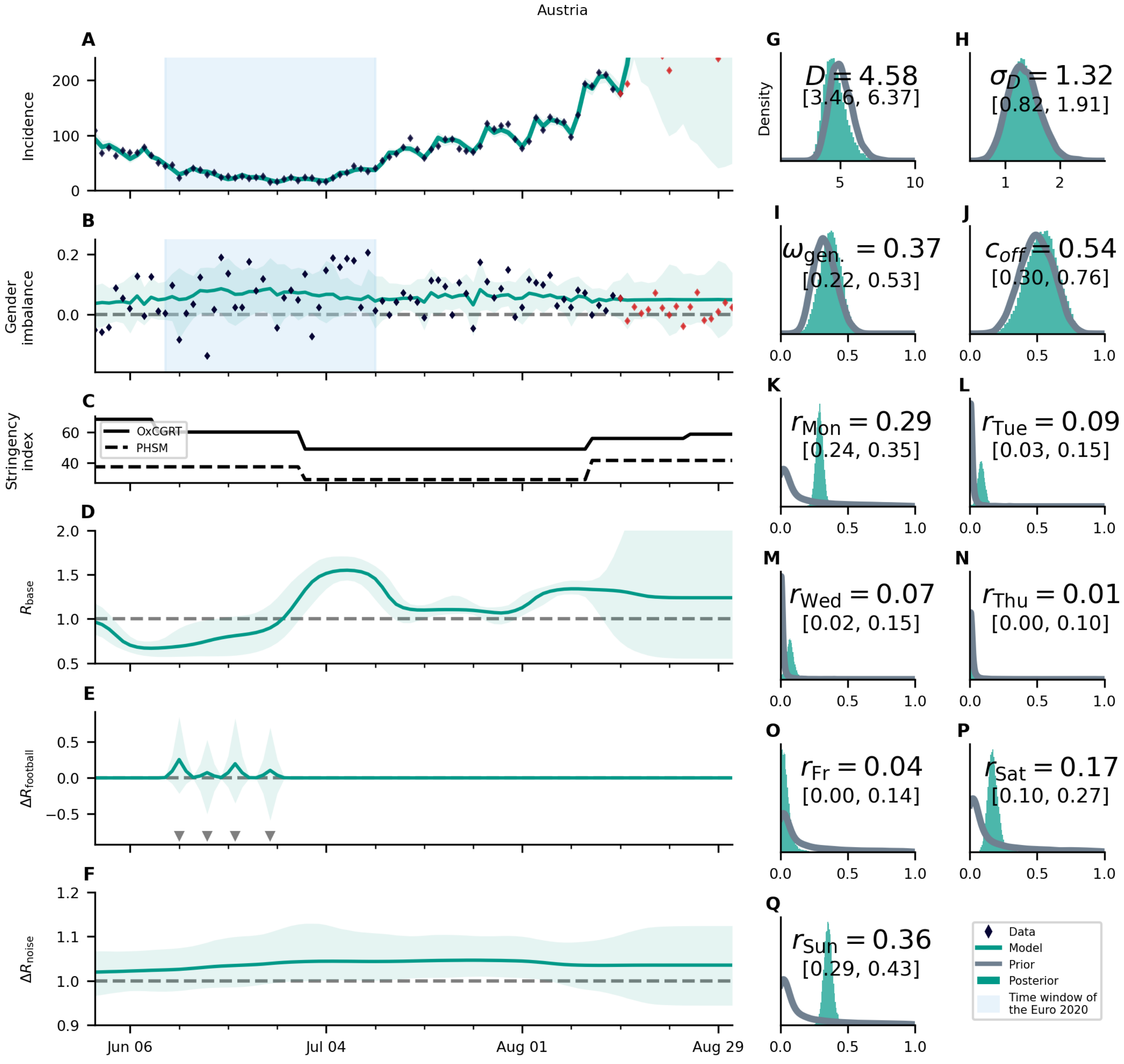


Supplementary Figure S25: **Overview of the posterior for Austria.** For an explanation of the panel structure, see supplementary Fig. S24. Austria shows a low significance for assigning cases to matches. The increase of $R_{\text{base}}$ coincides with the relaxation of restrictions **C**, but the subsequent decrease is not explained. The turquoise shaded areas correspond to 95% credible intervals.

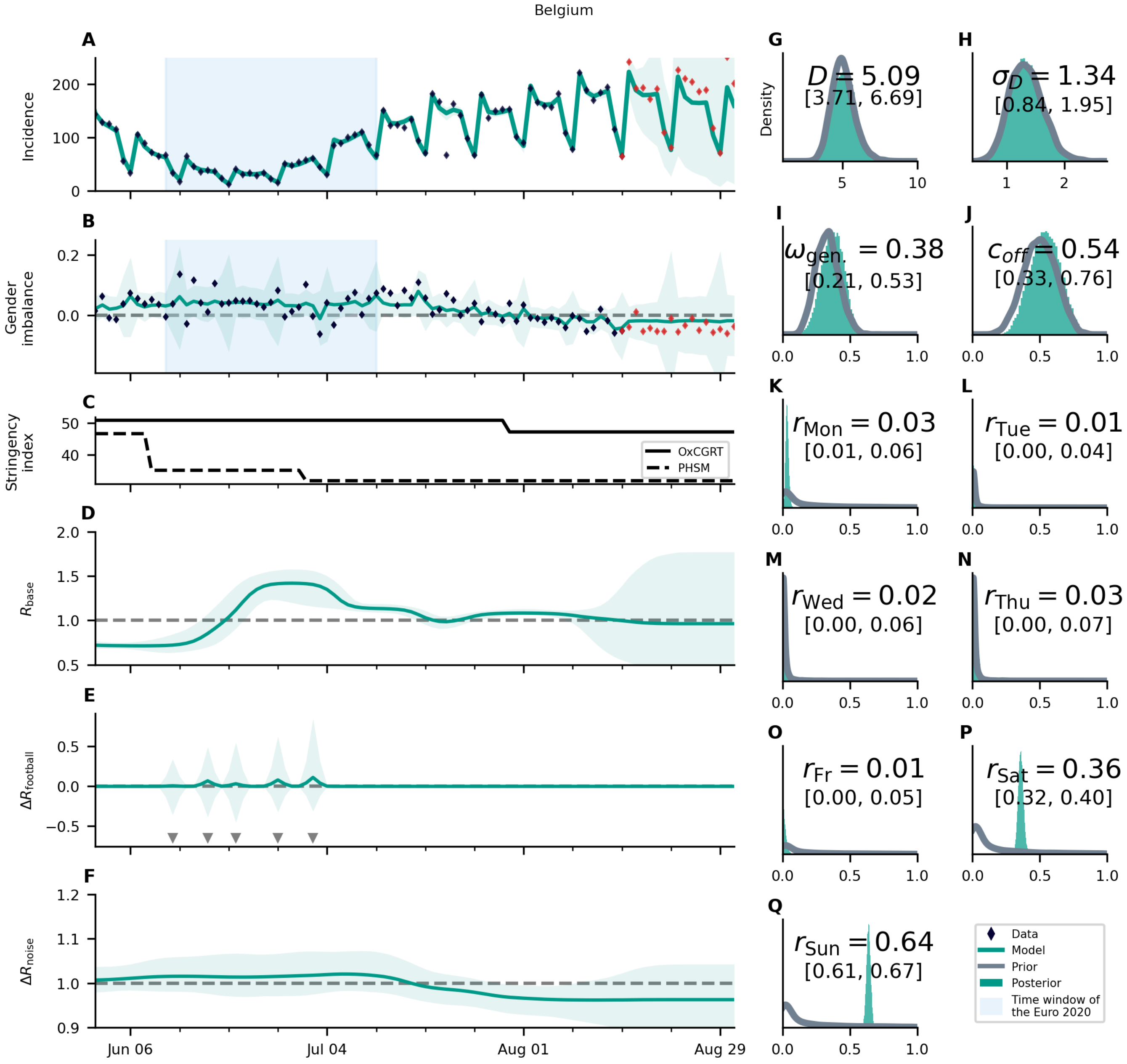


Supplementary Figure S26: **Overview of the posterior for Belgium.** For an explanation of the panel structure, see supplementary Fig. S24. Belgium shows a low significance for assigning cases to matches, but an intermittent increase of $R_{\text{base}}$ during the championship. The turquoise shaded areas correspond to 95% credible intervals.

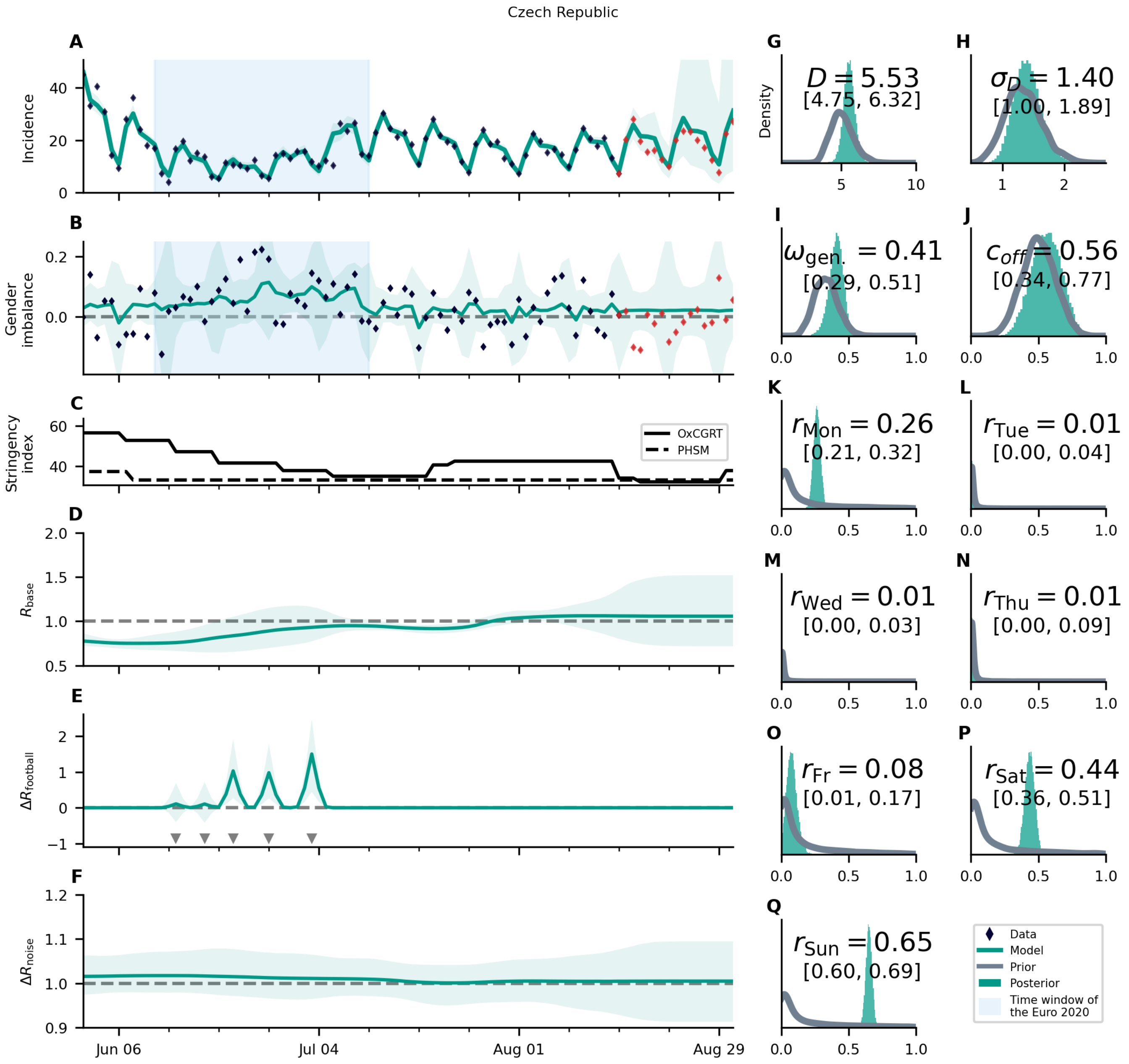


Supplementary Figure S27: **Overview of the posterior for the Czech Republic.** For an explanation of the panel structure, see supplementary Fig. S24. The overall incidence is relatively low, which increases the noisiness of the data. This is especially apparent in the gender imbalance (**B**). The base reproduction number is slowly increasing during the analyzed time-period, which can be partially explained by a decrease of the stringency index (**C**). The match effects are greater for later matches, beginning from the last group match until the quarterfinals (**E**), which is the expected variation. The turquoise shaded areas correspond to 95% credible intervals.

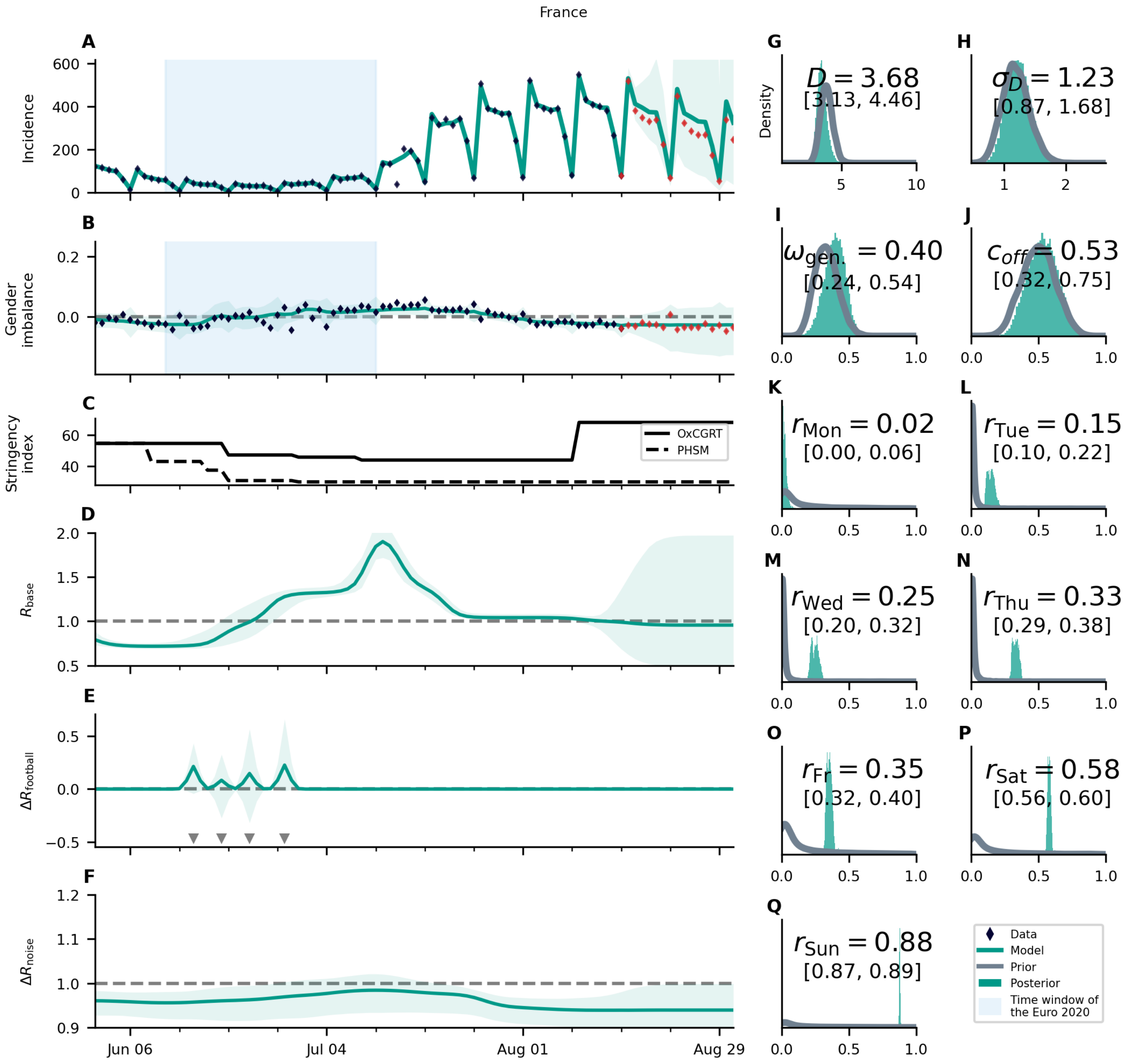


Supplementary Figure S28: **Overview of the posterior for France.** For an explanation of the panel structure, see supplementary Fig. S24. France shows a very pronounced increase of $R_{base}$ over the course of the championship and a very small fraction of cases assigned to matches of the French team. This hints at a rather gender-neutral effect of match-induced infections in France, in agreement with the results shown in Fig. S15. The peak of $R_{base}$ occurs on July 11th when clubs etc re-opened. It is unclear why the base reproduction number decreases this much again afterwards. The turquoise shaded areas correspond to 95% credible intervals.

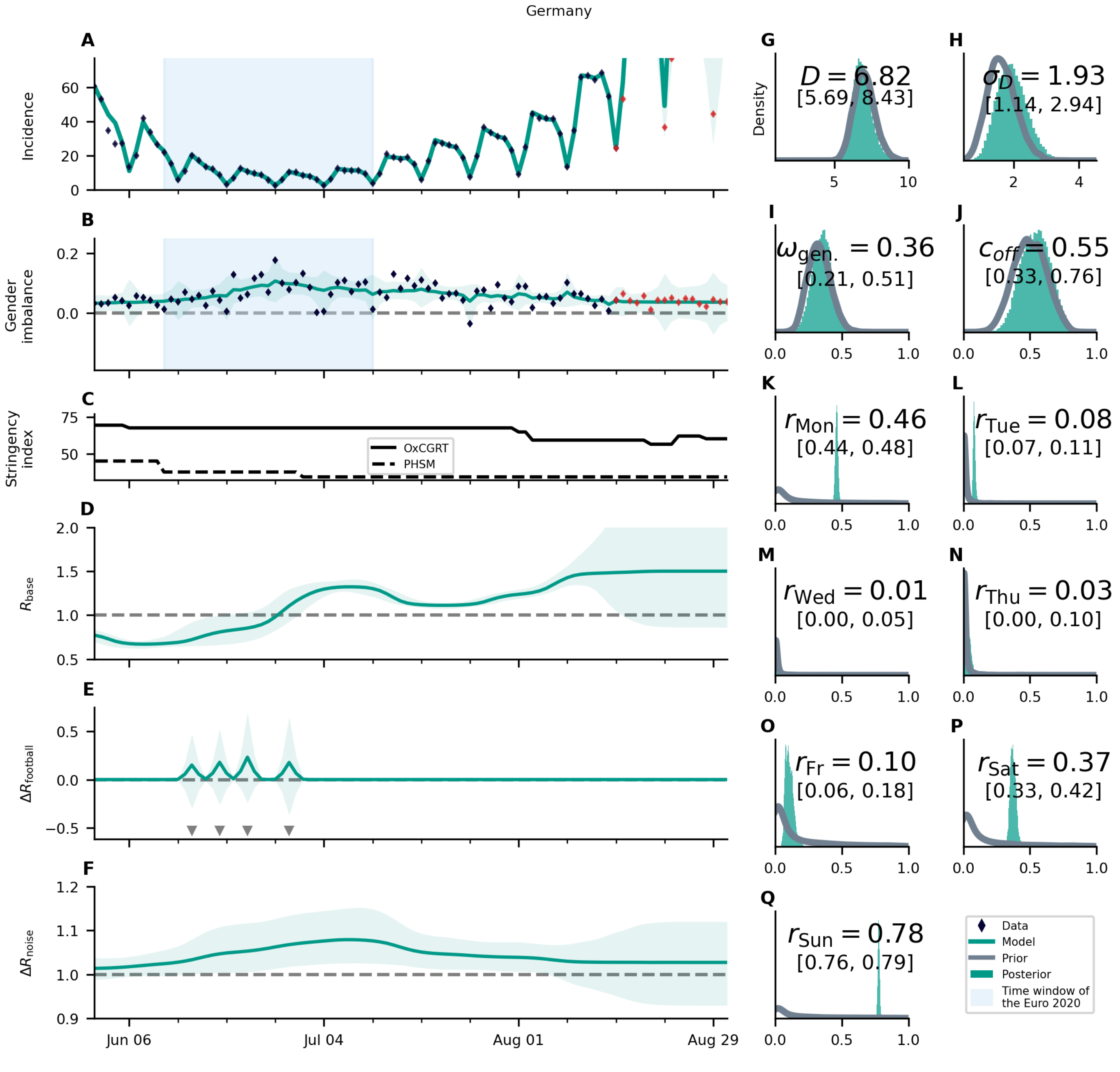


Supplementary Figure S29: **Overview of the posterior for Germany.** For an explanation of the panel structure, see supplementary Fig. S24. Germany shows an increase of $R_{base}$ and of the gender imbalance over the course of the championship (**B**). It might be the case that the Euro 2020 contribution is not tightly tied to matches of the German team, prohibiting the model to explain the observed gender imbalance via the individual matches (**E**), leading to an increase of $\Delta R_{\mathrm{noise}}$ instead (**F**). The turquoise shaded areas correspond to 95% credible intervals.

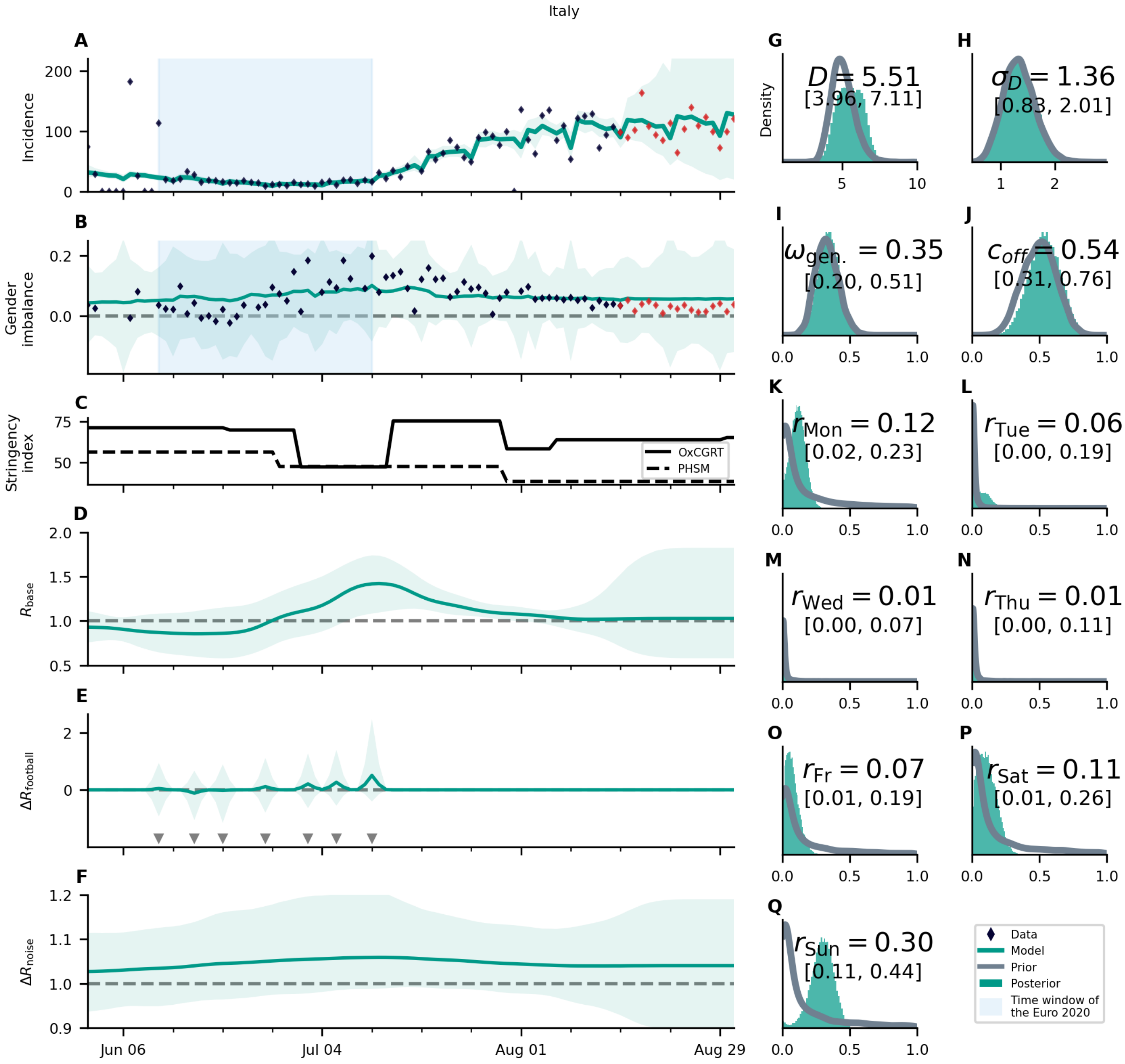


Supplementary Figure S30: **Overview of the posterior for Italy.** For an explanation of the panel structure, see supplementary Fig. S24. Italy is one of the countries where an intermittent increase in $R_{\text{base}}$ is observed (**D**). The development of the base reproduction number also coincides well with the relaxations and reinstatement of restrictions (**C**). Match-related football effects are not clearly visible (**E**). The turquoise shaded areas correspond to 95% credible intervals.

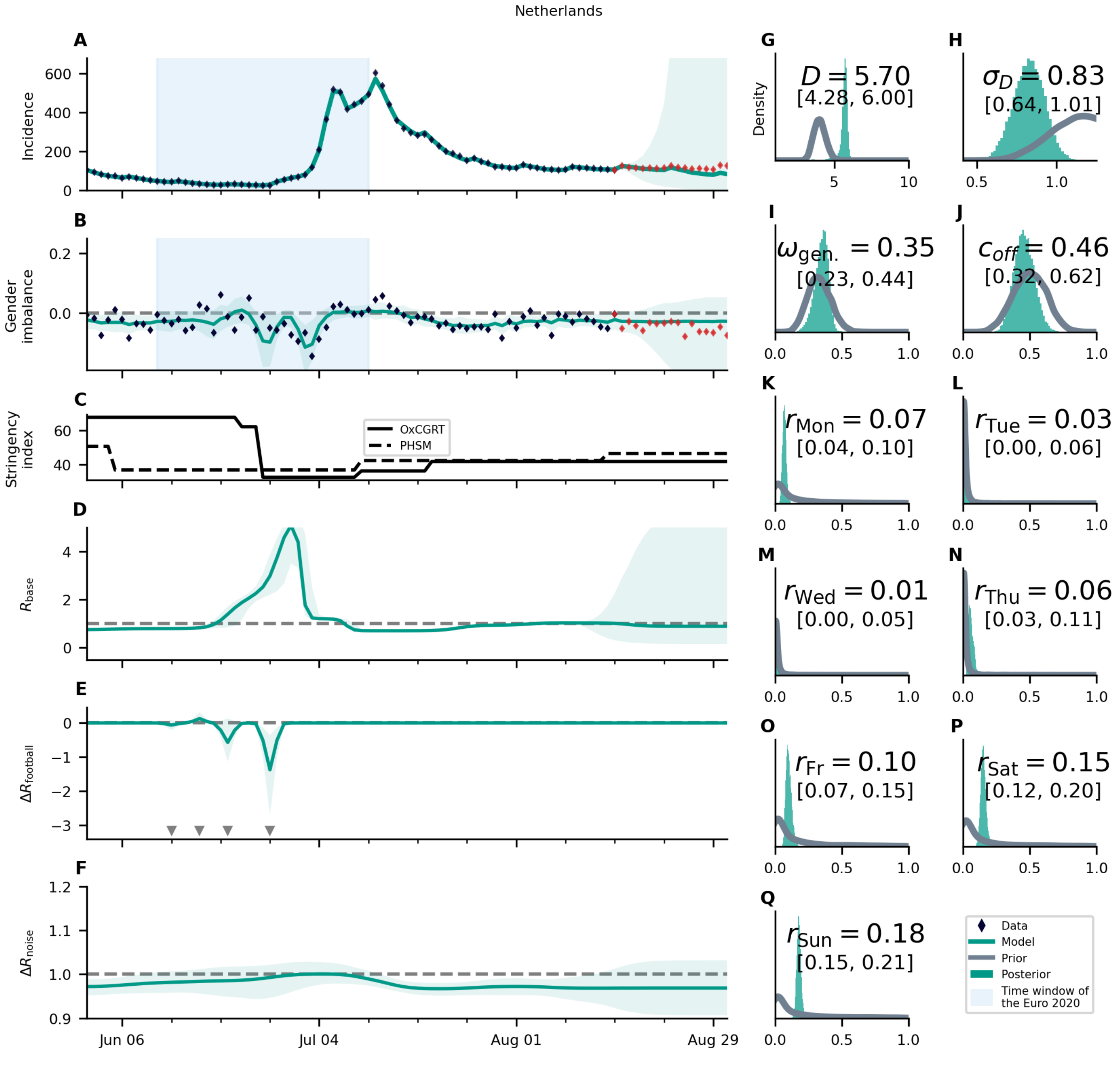


Supplementary Figure S31: **Overview of the posterior for the Netherlands.** For an explanation of the panel structure, see supplementary Fig. S24. The country wide "freedom day" on June 26th [10] is clearly visible in the incidence numbers **A** as well as the posterior base reproduction number **B**. Its effects overshadow possible effects from the Euro 2020 and we removed this country from subsequent analyses. The turquoise shaded areas correspond to 95% credible intervals.

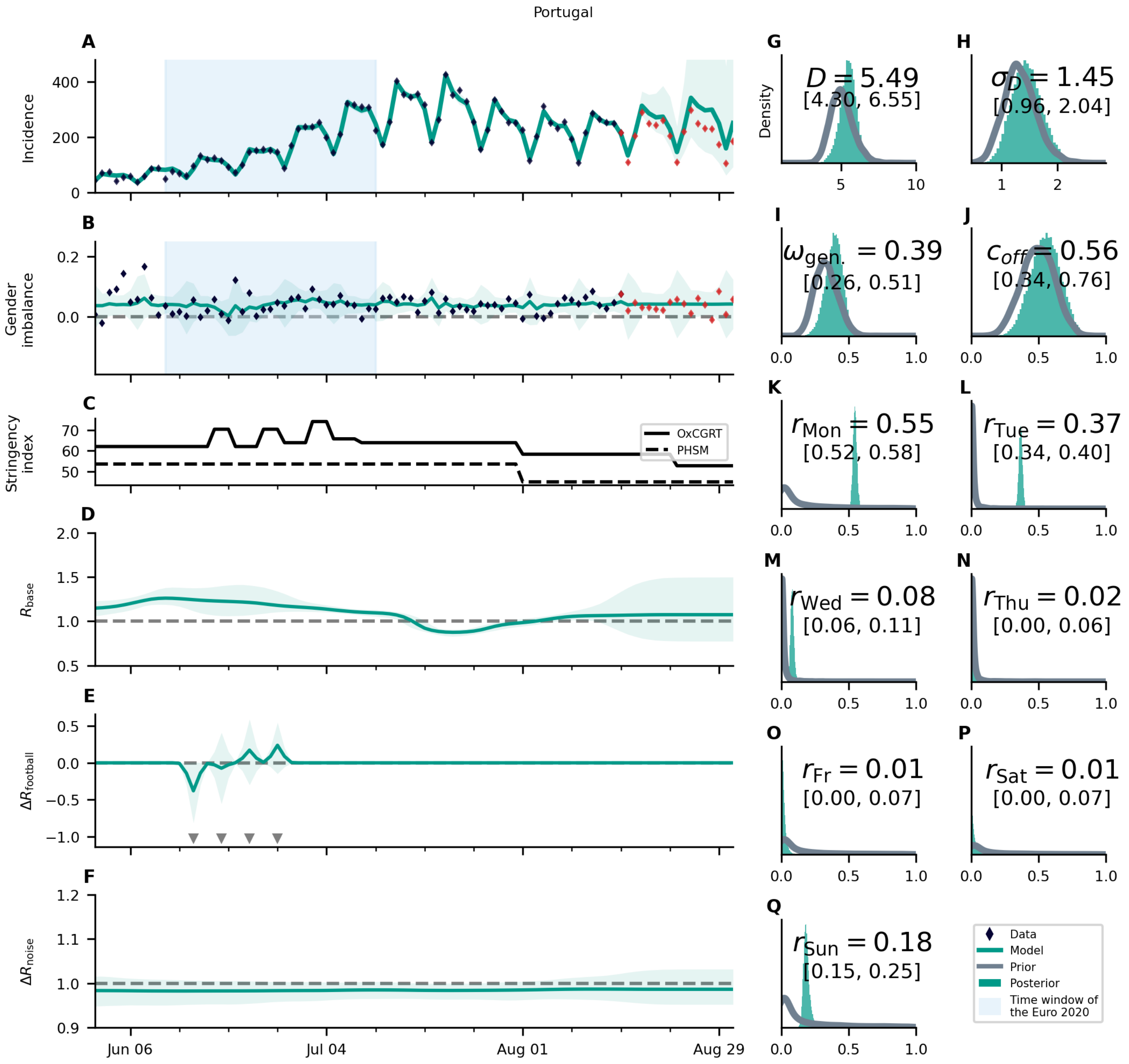


Supplementary Figure S32: **Overview of the posterior for Portugal.** For an explanation of the panel structure, see supplementary Fig. S24. Together with England, Portugal has the highest $R_{base}$ before the championship. It is the only country in which a decrease of $R_{base}$ over the course of the championship is observed. The fact that $R_{base}$ remains low after the championship could be a hint that the possible increase of cases due to the Euro 2020 in Portugal is small compared to the reduction stemming from unrelated changes. The turquoise shaded areas correspond to 95% credible intervals.

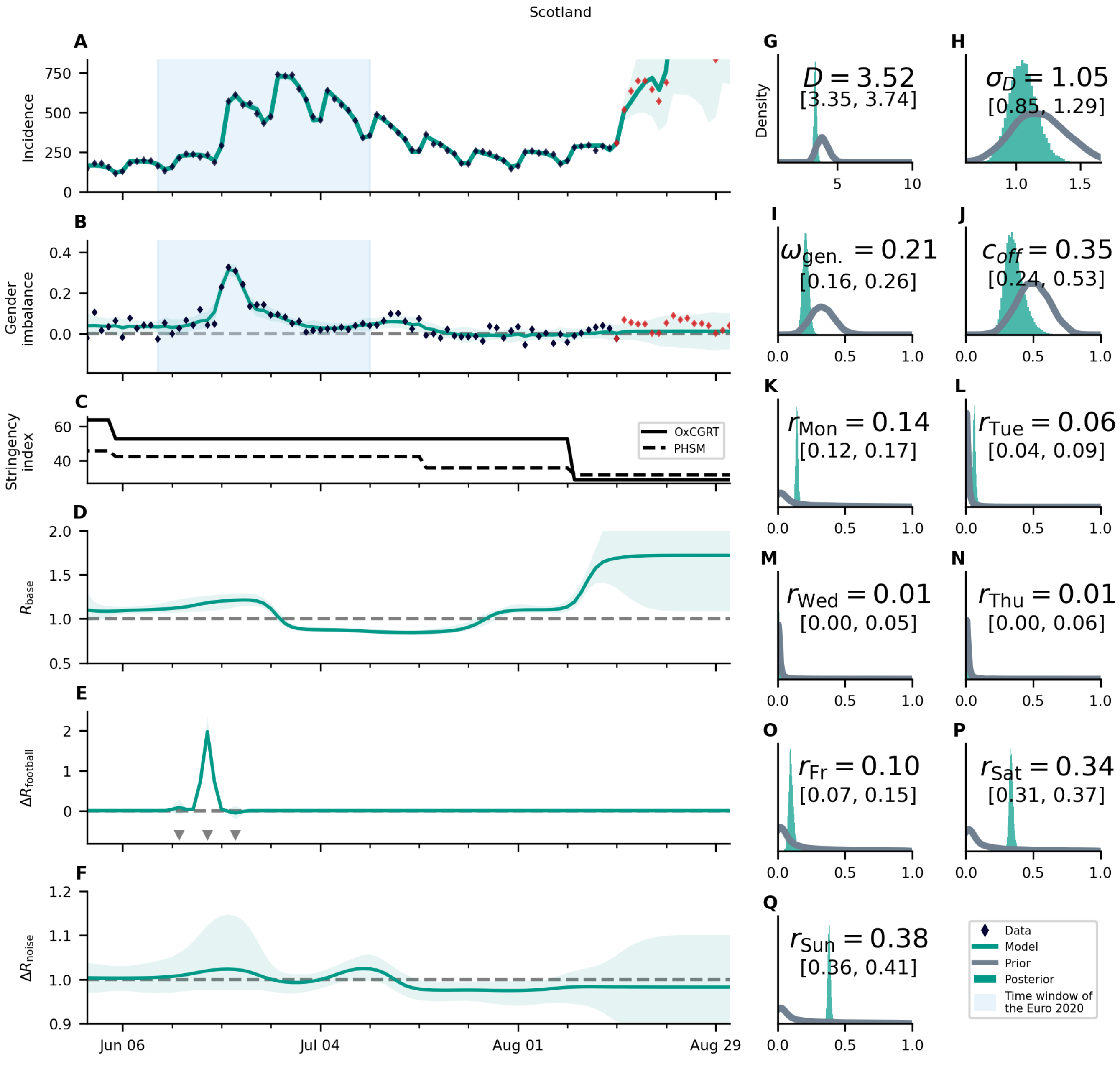


Supplementary Figure S33: **Overview of the posterior for Scotland.** For an explanation of the panel structure, see supplementary Fig. S24. Scotland is the country with the most significant effect of a single match, in this case against England. While this is in full agreement press reports (see also Fig. S20), the prior assumption of an exceptional large effect of this game is not built into the model. This clear association, thus, is a successful validation of the model functionality. The relaxation of governmental restrictions on August 9th is also well reflected in the development of the base reproduction number. The turquoise shaded areas correspond to 95% credible intervals.

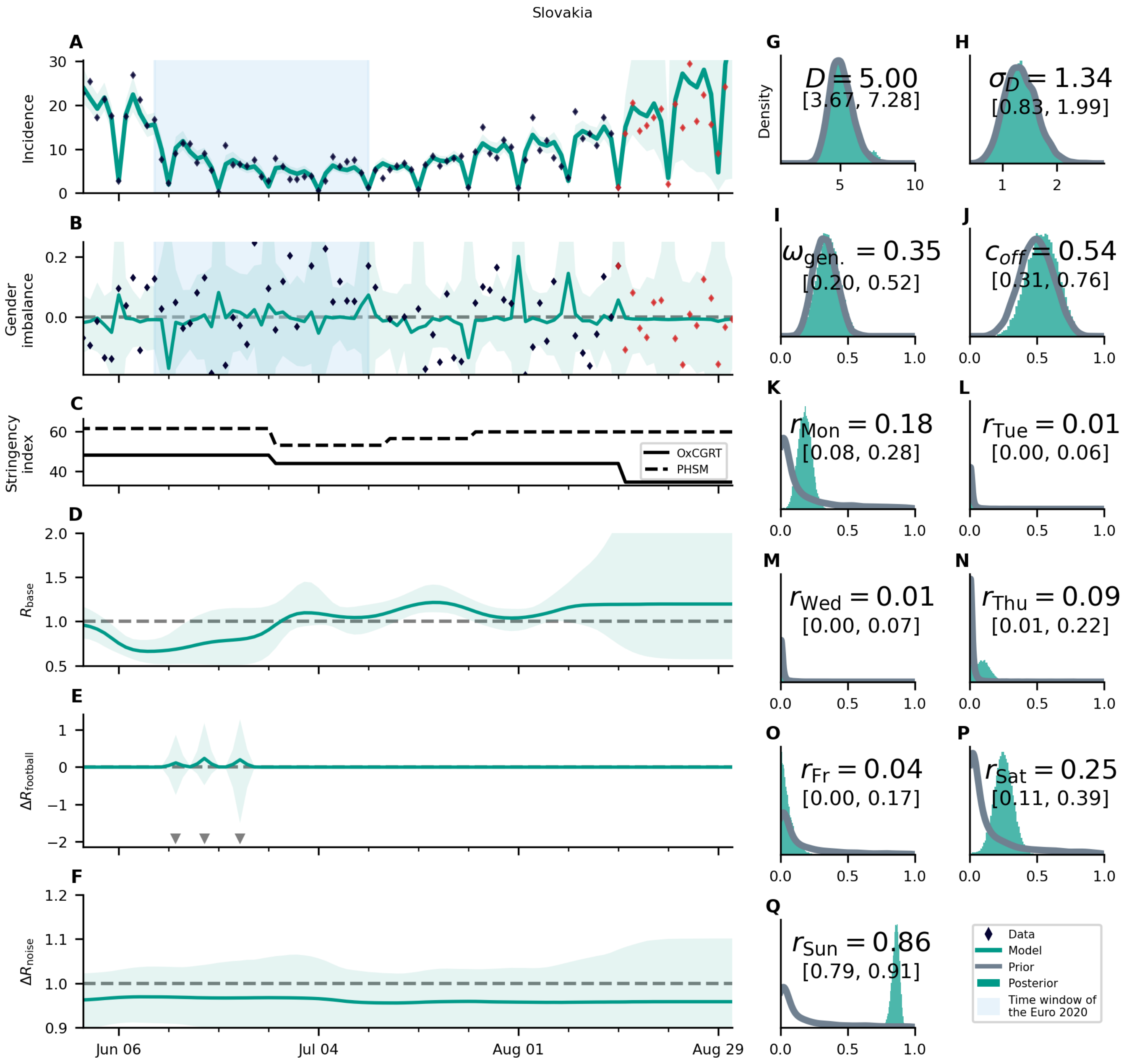


Supplementary Figure S34: **Overview of the posterior for Slovakia.** For an explanation of the panel structure, see supplementary Fig. S24. Hardly any significant effects, apart from a small but long-lasting increase in $R_{base}$, are observed. The turquoise shaded areas correspond to 95% credible intervals.

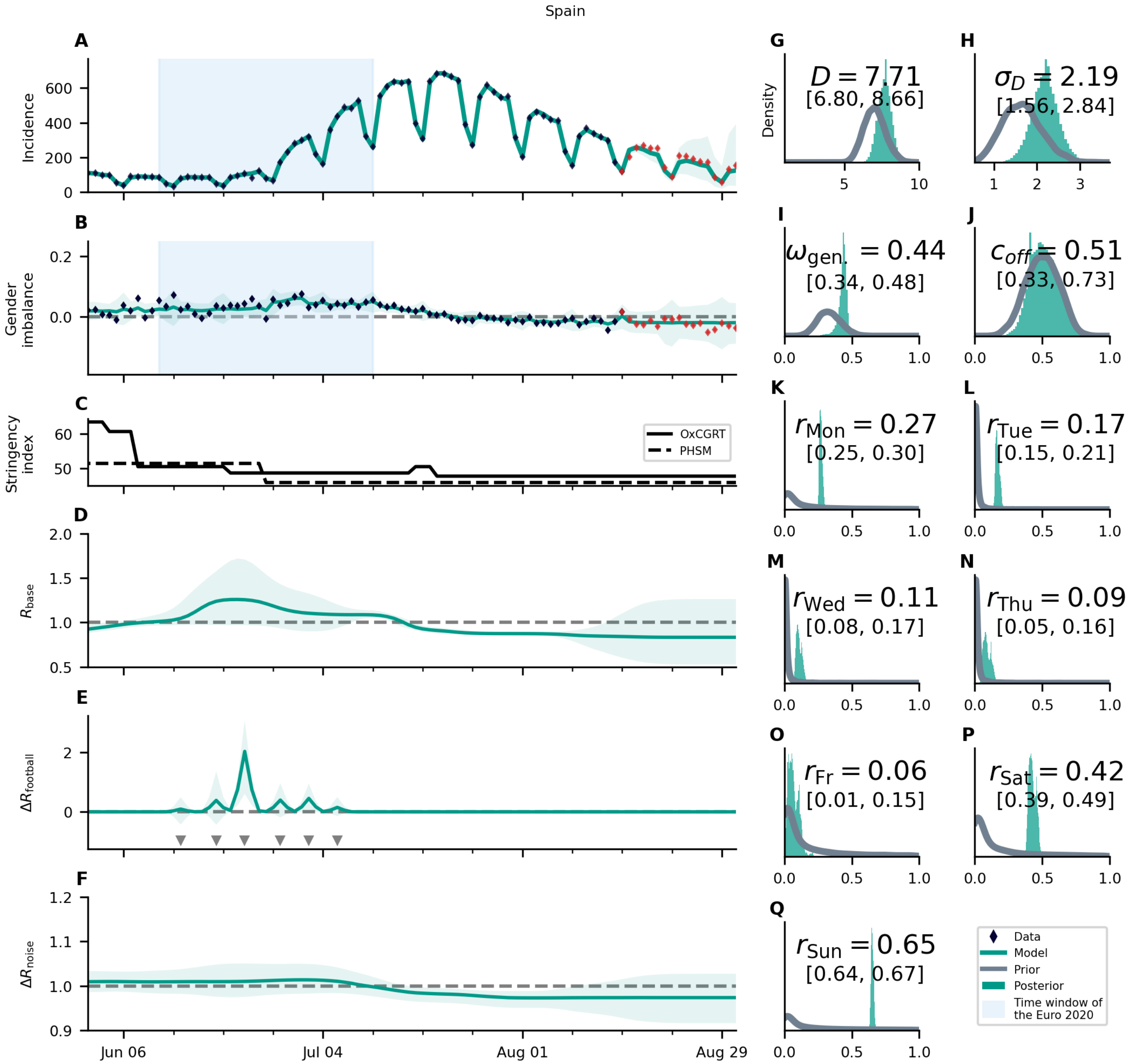


Supplementary Figure S35: **Overview of the posterior for Spain.** For an explanation of the panel structure, see supplementary Fig. S24. The national state of emergency ended in Spain on June 21st, in the middle of the championship. The model has therefore some difficulty to separate the effect of the relaxation of restrictions and the one of the matches, which translates into wide credible intervals in $R_{\text{base}}$ (**C**) and $\Delta R_{\text{football}}$ (**D**). The turquoise shaded areas correspond to 95% credible intervals.

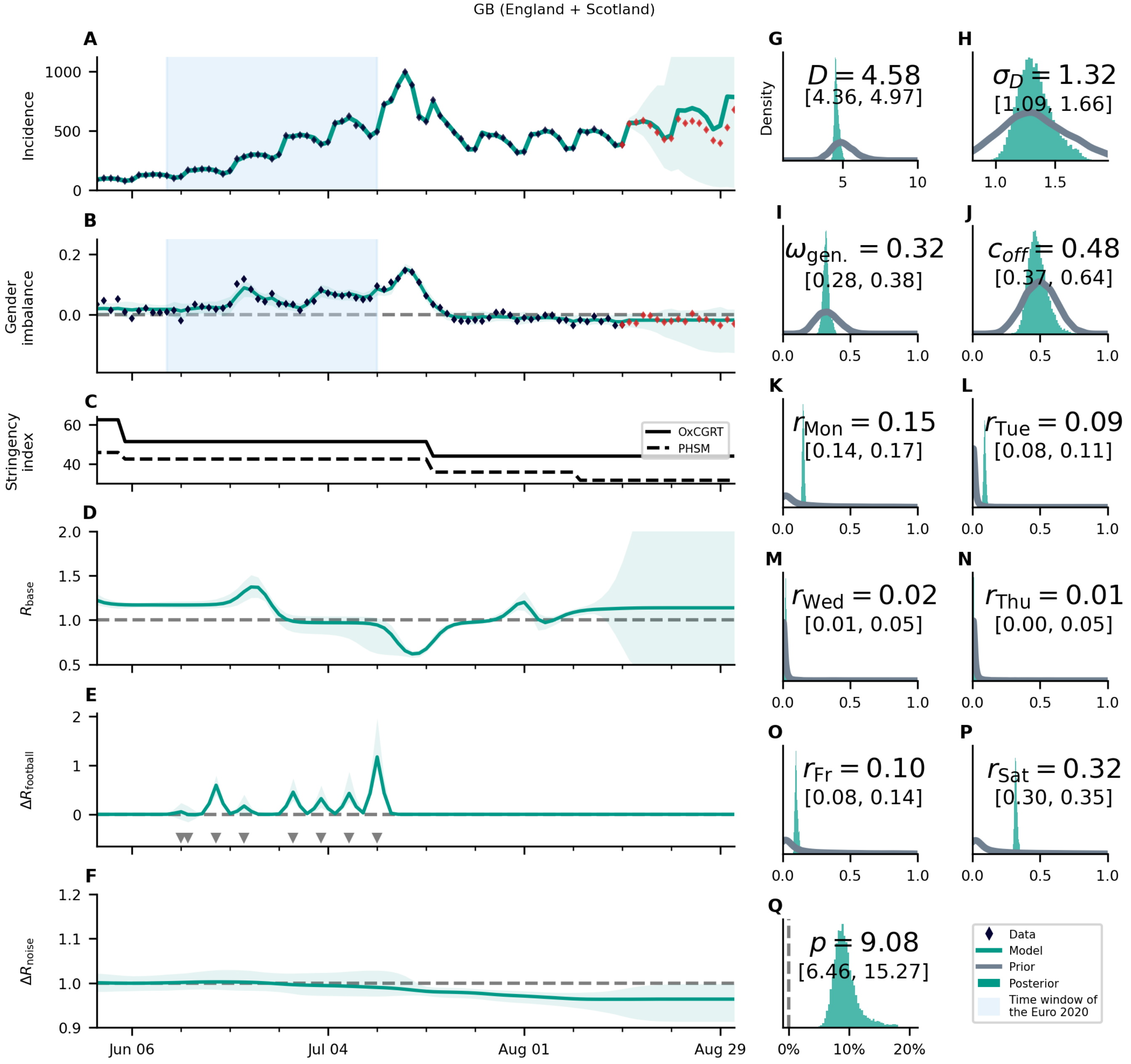


Supplementary Figure S36: **Overview of the posterior for the combined data of England and Scotland** For an explanation of the panel structure, see supplementary Fig. S24. The turquoise shaded areas correspond to 95% credible intervals.

## S4.6 Chain mixing of selected parameters

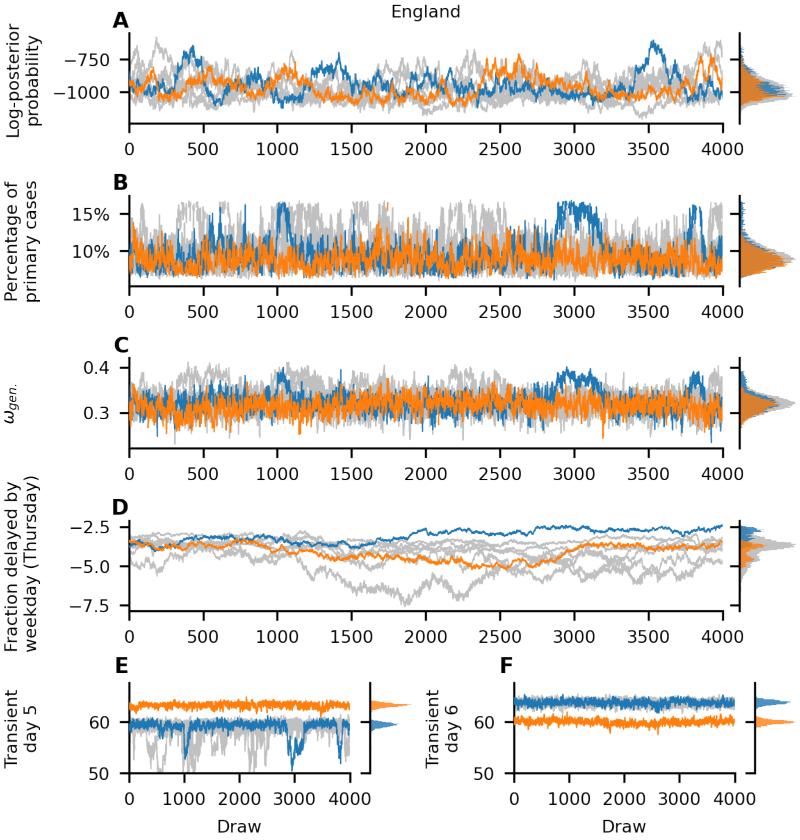


Supplementary Figure S37: **Chain mixing of selected parameters for England** Here we plot the unnormalized log-posterior probability (**A**) and selected parameters (**B** – **F**) as function for each draw and MCMC chain. Orange and blue depict two chains with the highest between-chain variance, the two least converging chains. The gray lines and histogram represent the ensemble of all chains. For our parameters of interest (**B**, **C**) the posterior distribution mixes well, even if the individual chains do not mix well in some other parameters (**D** – **F**), indicating that despite the degeneracy of some parameters, the inference of our parameters of interest is not affected. Panel **D** is a plot of the parameter with the worst mixing (the highest $\mathcal{R}$-hat value). Panels **E** and **F** show that the non-converging behavior can be explained as the exchange between two nearly degenerate solutions in two of the auxiliary parameters.

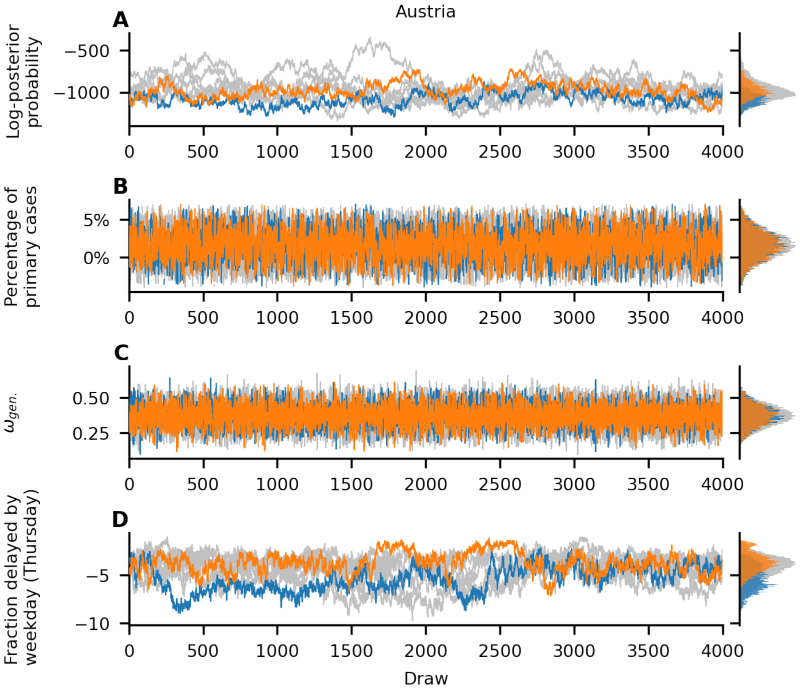


Supplementary Figure S38: **Chain mixing of selected parameters for Austria** Here the fraction of cases delayed by weekday on Thursdays is the parameter with the highest $\mathcal{R}$-hat values as seen in panel **D**. For a further detailed description of the panels see supplementary Fig. S37.

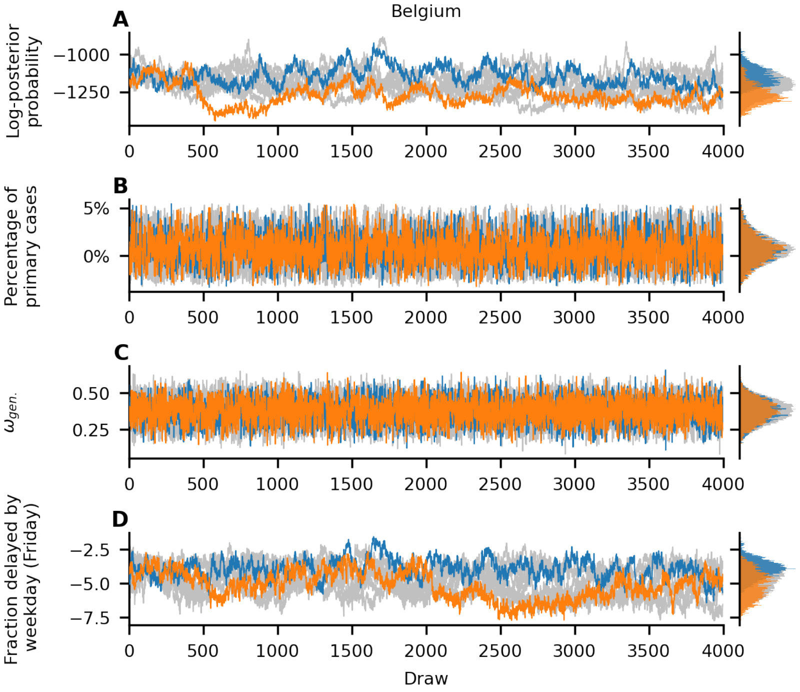


Supplementary Figure S39: **Chain mixing of selected parameters for Belgium** Here the fraction of cases delayed by weekday on Fridays is the parameter with the highest $\mathcal{R}$-hat values as seen in panel **D**. For a further detailed description of the panels see supplementary Fig. S37.

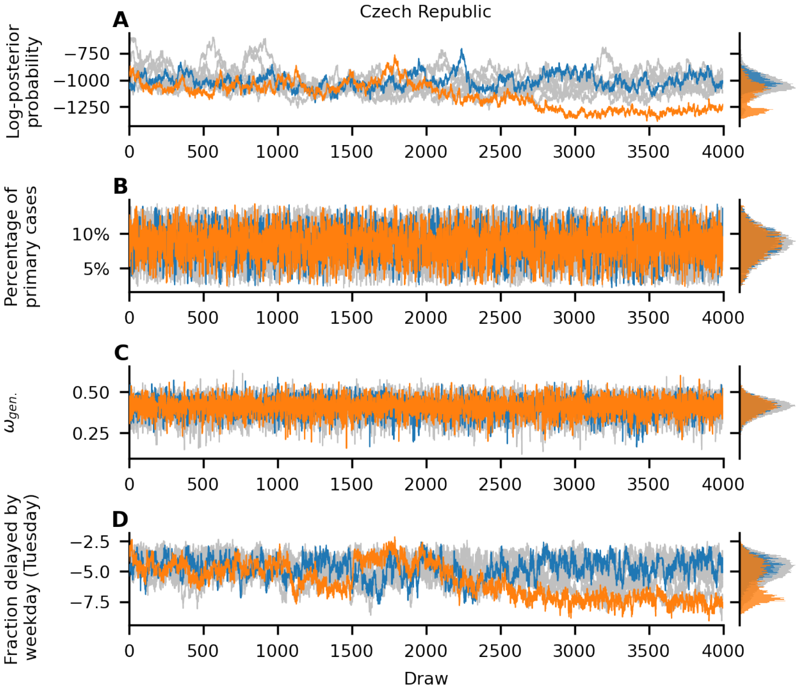


Supplementary Figure S40: **Chain mixing of selected parameters for Czech Republic** Here the fraction of cases delayed by weekday on Thursdays is the parameter with the highest $\mathcal{R}$-hat values as seen in panel **D**. For a further detailed description of the panels see supplementary Fig. S37.

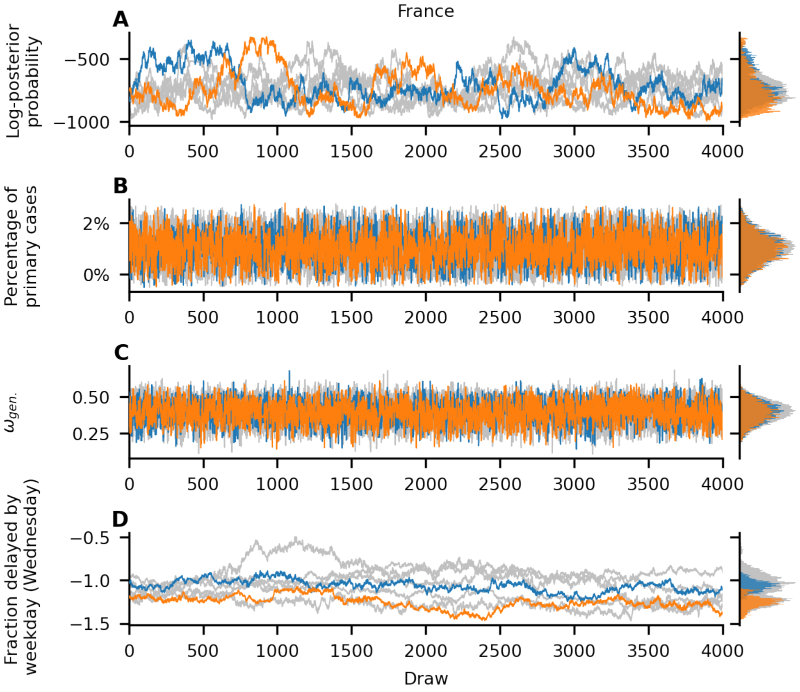


Supplementary Figure S41: **Chain mixing of selected parameters for France** Here the fraction of cases delayed by weekday on Wednesdays is the parameter with the highest $\mathcal{R}$-hat values as seen in panel **D**. For a further detailed description of the panels see supplementary Fig. S37.

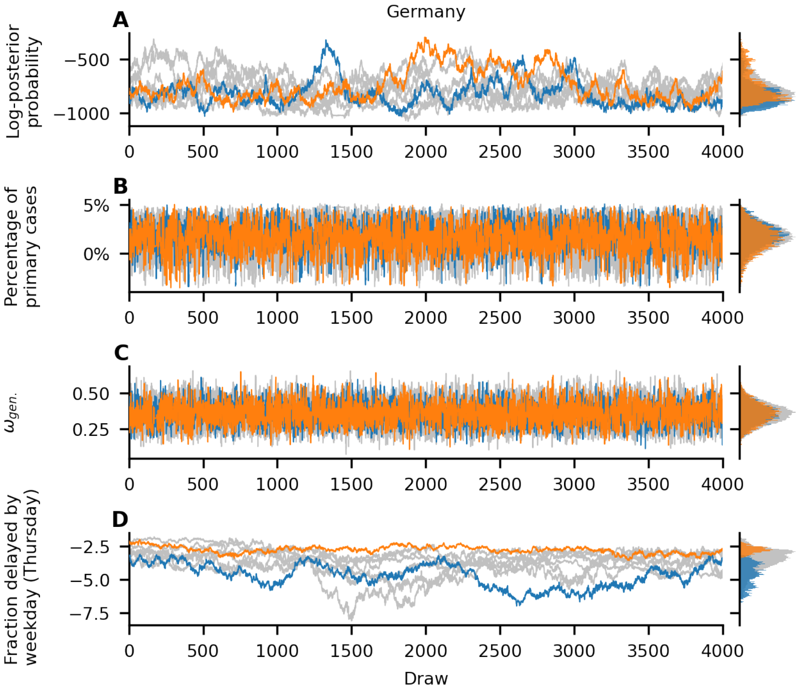


Supplementary Figure S42: **Chain mixing of selected parameters for Germany** Here the fraction of cases delayed by weekday on Thursdays is the parameter with the highest $\mathcal{R}$-hat values as seen in panel **D**. For a further detailed description of the panels see supplementary Fig. S37.

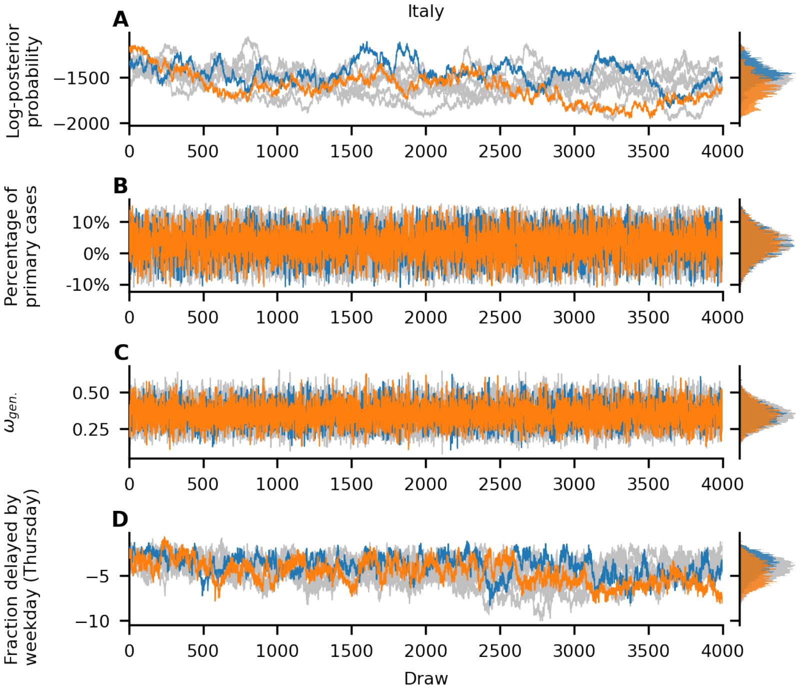


Supplementary Figure S43: **Chain mixing of selected parameters for Italy** Here the fraction of cases delayed by weekday on Thursdays is the parameter with the highest $\mathcal{R}$-hat values as seen in panel **D**. For a further detailed description of the panels see supplementary Fig. S37.

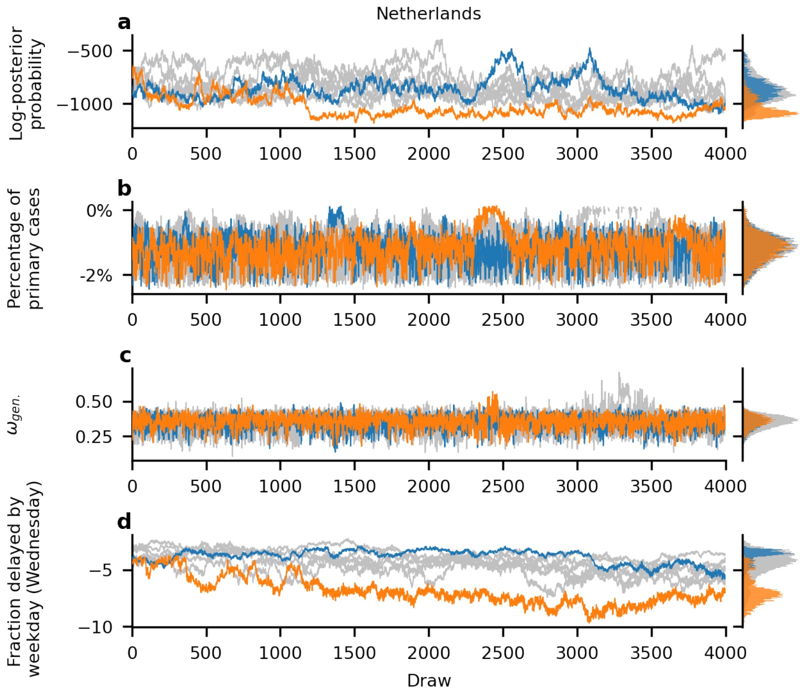


Supplementary Figure S44: **Chain mixing of selected parameters for Portugal** Here the fraction of cases delayed by weekday on Wednesdays is the parameter with the highest $\mathcal{R}$-hat values as seen in panel **D**. For a further detailed description of the panels see supplementary Fig. S37.

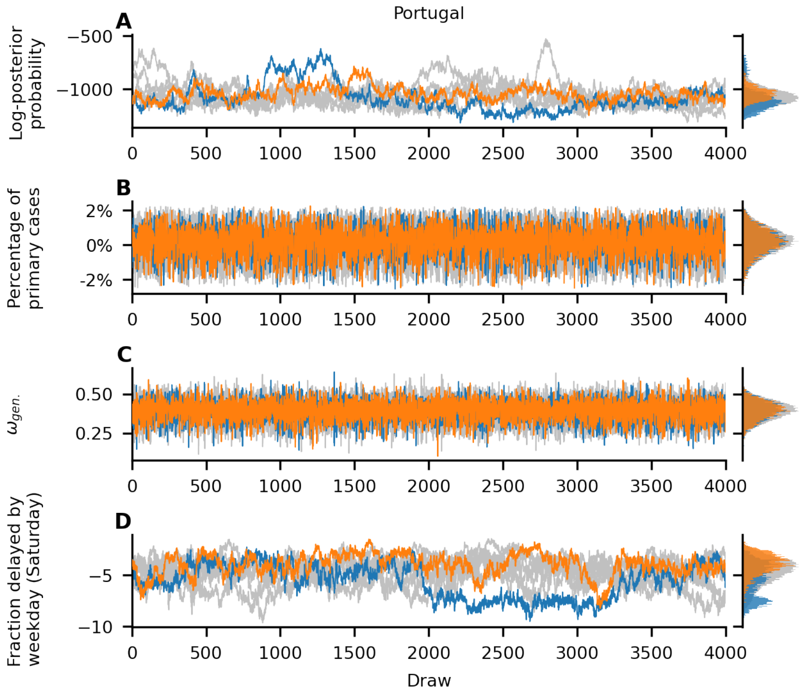


Supplementary Figure S45: **Chain mixing of selected parameters for Portugal** Here the fraction of cases delayed by weekday on Saturdays is the parameter with the highest $\mathcal{R}$-hat values as seen in panel **D**. For a further detailed description of the panels see supplementary Fig. S37.

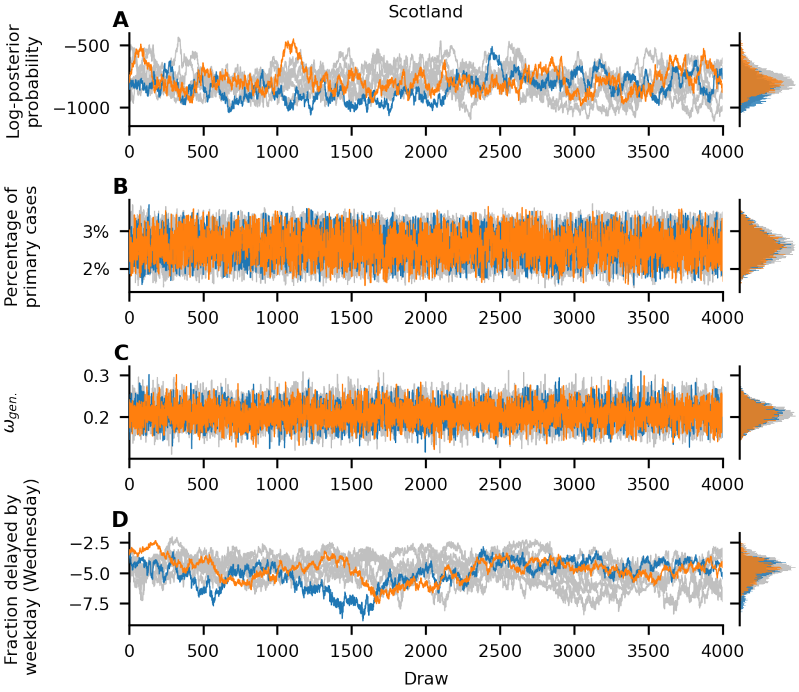


Supplementary Figure S46: **Chain mixing of selected parameters for Scotland** Here the fraction of cases delayed by weekday on Wednesdays is the parameter with the highest $\mathcal{R}$-hat values as seen in panel **D**. For a further detailed description of the panels see supplementary Fig. S37.

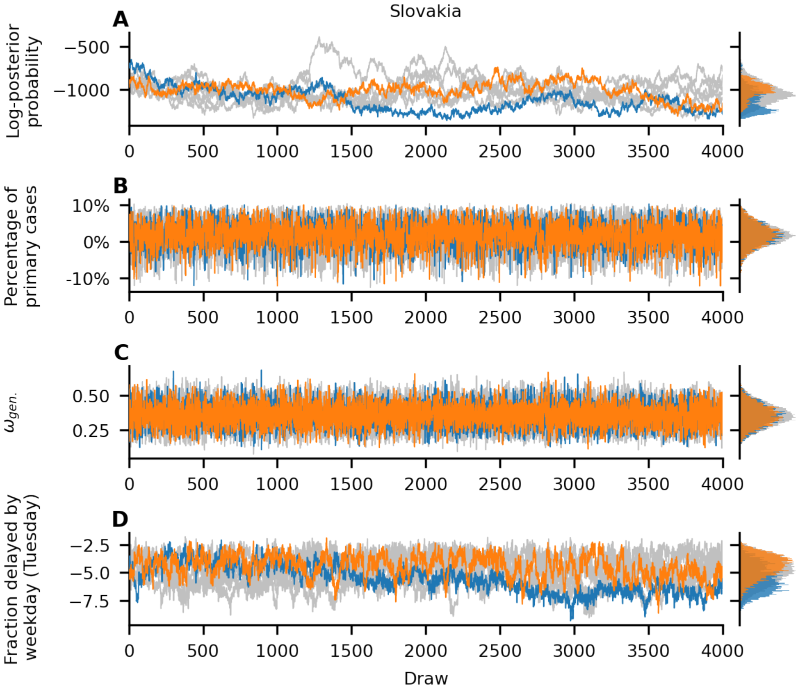


Supplementary Figure S47: **Chain mixing of selected parameters for Slovakia** Here the fraction of cases delayed by weekday on Thursdays is the parameter with the highest $\mathcal{R}$-hat values as seen in panel **D**. For a further detailed description of the panels see supplementary Fig. S37.

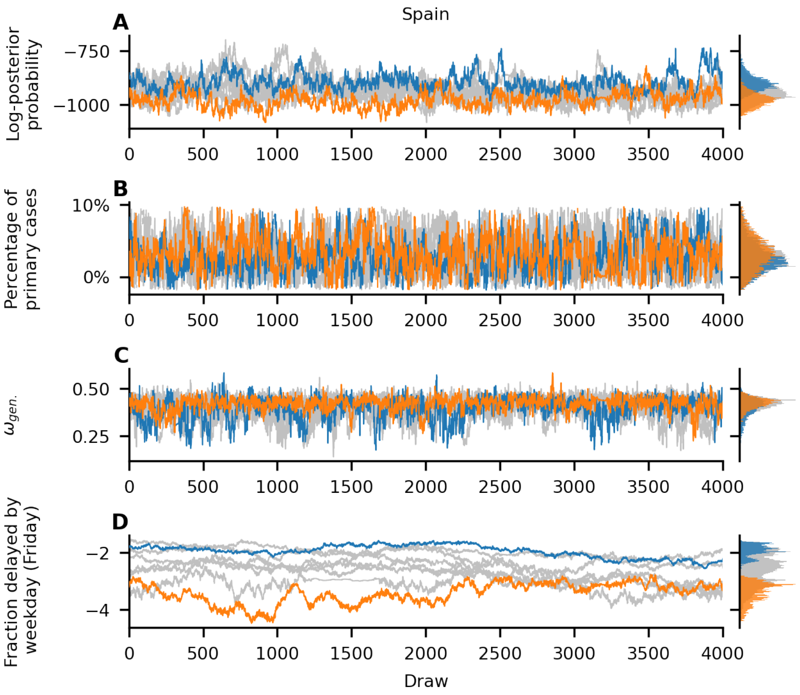


Supplementary Figure S48: **Chain mixing of selected parameters for Spain** Here the fraction of cases delayed by weekday on Fridays is the parameter with the highest $\mathcal{R}$-hat values as seen in panel **D**. For a further detailed description of the panels see supplementary Fig. S37.

## Supplementary References

[1] T. Riffe, E. Acosta, Data Resource Profile: COVerAGE-DB: a global demographic database of COVID-19 cases and deaths, *International Journal of Epidemiology* **50**, 390–390f (2021).

[2] E. O.-O. Max Roser, Hannah Ritchie, J. Hasell, Coronavirus Pandemic (COVID-19), *Our World in Data* (2020). `https://ourworldindata.org/coronavirus`, (Europe, America, and Oceania and Asia).

[3] T. Hale, *et al.*, A global panel database of pandemic policies (Oxford COVID-19 Government Response Tracker), *Nature Human Behaviour* **5**, 529–538 (2021).

[4] A systematic approach to monitoring and analysing public health and social measures (PHSM) in the context of the COVID-19 pandemic: underlying methodology and application of the PHSM database and PHSM Severity Index (2020).

[5] COVID-19 Community Mobility Reports, `https://www.google.com/covid19/mobility/`.

[6] E. Dong, H. Du, L. Gardner, An interactive web-based dashboard to track COVID-19 in real time, *The Lancet Infectious Diseases* **20**, 533 - 534 (2020).

[7] Google Trends, `https://trends.google.de/trends`.

[8] M. Sharma, *et al.*, Understanding the effectiveness of government interventions against the resurgence of COVID-19 in Europe, *Nature communications* **12**, 1–13 (2021).

[9] S. Lagaert, H. Roose, The gender gap in sport event attendance in Europe: The impact of macro-level gender equality, *International Review for the Sociology of Sport* **53**, 533-549 (2018).

[10] Oxford COVID-19 Government Response Tracker, Blavatnik School of Government and University of Oxford, `https://covidtracker.bsg.ox.ac.uk/`.
