# Supplementary material for: Impact of the Euro 2020 championship on the spread of COVID-19
Source: Nat Commun. 2023 Jan 18;14:122. doi: 10.1038/s41467-022-35512-x (PMC9849464; doi:10.1038/s41467-022-35512-x)
Supplement: Supplementary file 3 — Reporting Summary [file 41467_2022_35512_MOESM3_ESM.pdf]

nature portfolio

Corresponding author(s): Philip Bechtle, Viola Priesemann

Last updated by author(s): Sebastian B. Mohr, Jonas Dehning

Reporting Summary

Nature Portfolio wishes to improve the reproducibility of the work that we publish. This form provides structure for consistency and transparency in reporting. For further information on Nature Portfolio policies, see our Editorial Policies and the Editorial Policy Checklist.

Statistics

For all statistical analyses, confirm that the following items are present in the figure legend, table legend, main text, or Methods section.

n/a	Confirmed	
☐	☒	The exact sample size (*n*) for each experimental group/condition, given as a discrete number and unit of measurement
☒	☐	A statement on whether measurements were taken from distinct samples or whether the same sample was measured repeatedly
☒	☐	The statistical test(s) used AND whether they are one- or two-sided<br>*Only common tests should be described solely by name; describe more complex techniques in the Methods section.*
☐	☒	A description of all covariates tested
☐	☒	A description of any assumptions or corrections, such as tests of normality and adjustment for multiple comparisons
☐	☒	A full description of the statistical parameters including central tendency (e.g. means) or other basic estimates (e.g. regression coefficient) AND variation (e.g. standard deviation) or associated estimates of uncertainty (e.g. confidence intervals)
☒	☐	For null hypothesis testing, the test statistic (e.g. *F*, *t*, *r*) with confidence intervals, effect sizes, degrees of freedom and *P* value noted<br>*Give P values as exact values whenever suitable.*
☐	☒	For Bayesian analysis, information on the choice of priors and Markov chain Monte Carlo settings
☐	☒	For hierarchical and complex designs, identification of the appropriate level for tests and full reporting of outcomes
☐	☒	Estimates of effect sizes (e.g. Cohen's *d*, Pearson's *r*), indicating how they were calculated

Our web collection on statistics for biologists contains articles on many of the points above.

Software and code

Policy information about availability of computer code

Data collection	For the collection of data we used only publicly available data. See SI 'S1 Data sources' for more information.
Data analysis	We use pymc3 version 3.11.2 for MCMC sampling and model definition. Our code is available at: https://github.com/Priesemann-Group/covid19_soccer (DOI: 10.5281/zenodo.7386313)

For manuscripts utilizing custom algorithms or software that are central to the research but not yet described in published literature, software must be made available to editors and reviewers. We strongly encourage code deposition in a community repository (e.g. GitHub). See the Nature Portfolio guidelines for submitting code & software for further information.

Data

Policy information about availability of data

All manuscripts must include a data availability statement. This statement should provide the following information, where applicable:

- Accession codes, unique identifiers, or web links for publicly available datasets
- A description of any restrictions on data availability
- For clinical datasets or third party data, please ensure that the statement adheres to our policy

The data from our model runs, i.e., from the sampling is available on G-node https://gin.g-node.org/semohr/covid19_soccer_data. The daily case numbers stratified by age and gender were acquired from the local health authorities (see also Supplementary Information "S1 Data sources') from the following sources: https://www.arcgis.com/home/item.html?id=f10774f1c63e40168479a1feb6c7ca74 , https://www.data.gouv.fr/fr/datasets/taux-dincidence-de-lepidemie-de-

covid-19 , https://coronavirus.data.gov.uk/details/download , https://covid19-dashboard.ages.at , https://epistat.wiv-isp.be/covid , https://onemocneni-aktualne.mzcr.cz/covid-1 , https://data.rivm.nl/covid-19 , https://www.coverage-db.org

Human research participants

Policy information about studies involving human research participants and Sex and Gender in Research.

Reporting on sex and gender	Our study takes into account the gender of people infected by SARS-CoV-2. However, only the aggregated count of reported cases is being used. We do not possess individual level data. The gender information has been collected by the health authorities of the European countries included in this study.
Population characteristics	We use publicly available data of the number of COVID-19 cases that doesn't include individual level data nor population characteristics except for the gender
Recruitment	No research participants were recruited
Ethics oversight	As we didn't recruit participants and used only publicly available data, no ethics oversight was present.

Note that full information on the approval of the study protocol must also be provided in the manuscript.

Field-specific reporting

Please select the one below that is the best fit for your research. If you are not sure, read the appropriate sections before making your selection.

☒ Life sciences ☐ Behavioural & social sciences ☐ Ecological, evolutionary & environmental sciences

For a reference copy of the document with all sections, see nature.com/documents/nr-reporting-summary-flat.pdf

Life sciences study design

All studies must disclose on these points even when the disclosure is negative.

Sample size	We analyze all countries which reported the gender of COVID-19 cases on a daily basis and participated in the Euro 2020.
Data exclusions	We excluded The Netherlands from the average effect and correlation calculations because the relaxation of governmental interventions occured within the period of the Championship and had a large effect on case numbers and their gender imbalance.
Replication	We conducted extensive robustness tests of our results, see Supplementary Figures S11-S19.
Randomization	We performed no randomization ourself. Our study has a random part because the dates and participant countries of the Euro 2020 matches depended on the draw performed by the UEFA and the subsequent success of the teams during the championship. This allocation of matches is nearly independent of the state of the pandemic and therefore doesn't influence our results.
Blinding	There is no group allocation and thus no blinding.

Reporting for specific materials, systems and methods

We require information from authors about some types of materials, experimental systems and methods used in many studies. Here, indicate whether each material, system or method listed is relevant to your study. If you are not sure if a list item applies to your research, read the appropriate section before selecting a response.

Materials & experimental systems

n/a	Involved in the study	
☒	☐	Antibodies
☒	☐	Eukaryotic cell lines
☒	☐	Palaeontology and archaeology
☒	☐	Animals and other organisms
☒	☐	Clinical data
☒	☐	Dual use research of concern

Methods

n/a	Involved in the study	
☒	☐	ChIP-seq
☒	☐	Flow cytometry
☒	☐	MRI-based neuroimaging
